# Facile total synthesis of lysicamine and the anticancer activities of the Ru^II^, Rh^III^, Mn^II^ and Zn^II^ complexes of lysicamine

**DOI:** 10.18632/oncotarget.19584

**Published:** 2017-07-26

**Authors:** Jiao-Lan Qin, Ting Meng, Zhen-Feng Chen, Xiao-Li Xie, Qi-Pin Qin, Xiao-Ju He, Ke-Bin Huang, Hong Liang

**Affiliations:** ^1^ State Key Laboratory for Chemistry and Molecular Engineering of Medicinal Resources, School of Chemistry and Pharmacy, Guangxi Normal University, Guilin 541004, P. R. China

**Keywords:** lysicamine, metal complexes, antitumor activity, apoptosis

## Abstract

Lysicamine is a natural oxoaporphine alkaloid, which isolated from traditional Chinese medicine (TCM) herbs and has been shown to possess cytotoxicity to hepatocarcinoma cell lines. Reports on its antitumor activity are scarce because lysicamine occurs in plants at a low content. In this work, we demonstrate a facile concise total synthesis of lysicamine from simple raw materials under mild reaction conditions, and the preparation of the Ru(II), Rh(III), Mn(II) and Zn(II) complexes 1–4 of lysicamine (LY). All the compounds were fully characterized by elemental analysis, IR, ESI-MS, ^1^H and ^13^C NMR, as well as single-crystal X-ray diffraction analysis. Compared with the free ligand LY, complexes 2 and 3 exhibited superior *in vitro* cytotoxicity against HepG2 and NCI-H460. Mechanistic studies indicated that 2 and 3 blocked the cell cycle in the S phase by decreasing of cyclins A2/B1/D1/E1, CDK 2/6, and PCNA levels and increasing levels of p21, p27, p53 and CDC25A proteins. In addition, 2 and 3 induced cell apoptosis via both the caspase-dependent mitochondrial pathway and the death receptor pathway. *in vivo* study showed that 2 inhibited HepG2 tumor growth at 1/3 maximum tolerated dose (MTD) and had a better safety profile than cisplatin.

## INTRODUCTION

Many oxoaporphine alkaloids are derived from traditional Chinese medicine (TCM) herbs and exhibit various pharmacological properties, such as antiplatelet aggregation [1], anti-inflammation [2], antitubercular [3] and antitumor activities [4, 5], *etc*. These medicinally interesting compounds generally have very low content (<0.01%) in plants, and their biological activities are thus reported only scarcely.

Some research teams have devoted considerable efforts to synthesize oxoaporphine alkaloids [6–9]. Oxidation of aporphines is the easiest way to produce oxoaporphines, and this has allowed the synthesis of glaucine [10], diisopropylboldine [11], and (+)-boldine [12, 13]. However, the source aporphines have limited commercial supply. The total synthesis of oxoaporphines has been reported both in the early years [6–9] and recently [14–16]. The reported syntheses have clear disadvantages, including numerous reaction steps, long reaction time, harsh reaction conditions, complicated work-up procedures, extensive use of organic solvents, *etc*. Needless to say, a short and efficient route for the total synthesis of oxoaporphine alkaloids is urgently needed.

Because of the side effects of cisplatin in chemotherapy, non-platinum metal-based antitumor agents that are highly efficient and less toxic are sought after in recent decades [17, 18]. Among thousands of candidates, three ruthenium complexes, *i.e*., [ImH] [*trans*-RuCl_4_(DMSO)Im] (NAMI-A) [19], [ImH] [*trans*-RuCl_4_Im_2_] (KP1019) [20], and Na[*trans*-RuCl_4_Im_2_] (NKP-1339 or IT-139) [21] are currently tested in clinical trials as anticancer drugs. Besides, the complexes of Rh(III) [22–26], Zn(II) [27–29], and Mn(II) [30, 31] with potential antitumor activity have also been reported.

An increasing number of studies indicate that coordination compounds based on traditional Chinese medicines (TCMs) have excellent antitumor activity [32]. We previously reported on the antitumor activity of liriodenine, oxoglaucine, and oxoisoaporphine, and demonstrated that the efficacy was much higher in the liriodenine metal complexes (with Pt^II^, Ru^II^, Mn^II^, Fe^II^, Co^II^, and Zn^II^) [33, 34], oxoglaucine metal complexes (with Au^III^, Zn^II^, Co^II^, Mn^II^, Y^III^, and Dy^III^) [12, 13], and oxoisoaporphine metal complexes (with Ni^II^, Pd^II^ and Pt^II^) [35, 36] compared with that of the corresponding ligands.

Primary screening results revealed that lysicamine had cytotoxicity against hepatocarcinoma cell lines [37] and human adult T-cell leukemia/lymphoma (III) [38]. In this study, we demonstrate a very efficient route to synthesize lysicamine from simple starting materials under mild conditions. In addition, four metal complexes of lysicamine, *i.e*., [Ru(LY)Cl_2_(DMSO)_2_]·3H_2_O (**1**), [Rh(LY-OH)Cl_3_CH_3_OH] (**2**), [Mn(LY)_3_](ClO_4_)_2_·3CHCl_3_ (**3**), and [Zn(LY)_2_(ClO_4_)_2_] (**4**), were synthesized and fully characterized. The cytotoxicity and antitumor mechanism of these metal complexes were investigated. The *in vivo* antitumor efficacy of **2** was further evaluated in HepG2 xenograft nude mice models.

## RESULTS

### Total synthesis of lysicamine (LY)

**Figure 1:**
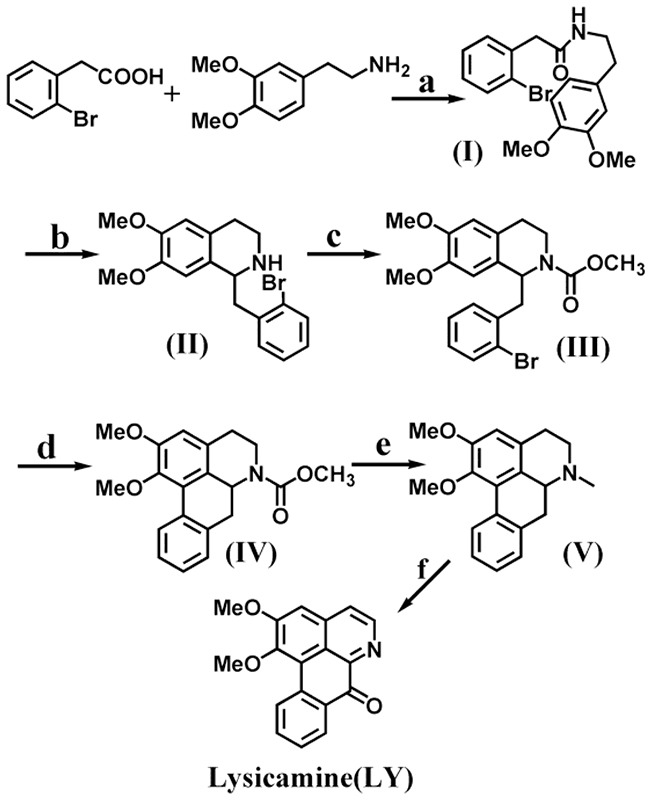
Synthetic routes of lysicamine (LY) Reagents and conditions are as follows: a: (i) SOCl_2_, CHCl_3_ (75 °C reflux, 2 h); (ii) CHCl_3_, NaHCO_3_ (ice-bath, 2 h); b: (i) POCl_3_, toluene (80 °C reflux, 3 h); (ii) (CH_3_COO)_3_BHNa, CHCl_3_ (room temperature, 1 h); c: Methyl chloroformate (ClCOOCH_3_), NaOH, CHCl_3_ (room temperature, 1 h); d: tricyclohexyl phosphine [P(cy)_3_], Pd(OAc)_2_, K_2_CO_3_, DMA (120 °C, N_2_,5 h); e: LiAlH_4_, THF (reflux, 6 h); f: Mn(Ac)_3_, glacial acetic acid (80 °C, 12 h).

Figure 1 shows the synthetic route, which started with commercially available starting materials, 2-bromophenylacetic acid and 3,4-dimethoxyphenethylamine. The amide (**I**) was obtained from amide coupling reaction. Specifically, 2-bromophenylacetic acid was treated with SOCl_2_ in CHCl_3_ to generate 2-bromophenylacetyl chloride, which in turn reacted with 3,4-dimethoxyphenethylamine in CHCl_3_ to give amide (**I**) as white needles in 85% yield. The Bischler−Napieralski reaction [39] was employed for the cyclization of (**I**) and furnish the tetrahydroisoquinolines (**II**). The intermediate imine was reduced by (CH_3_COO)_3_BHNa without further purification [40] to afford (**II**) in 82% yield.

Previously reports [41–43] often constructed the ring C of the aporphine nucleus through radical-initiated cyclization. However, direct radical cyclization of (**II**) was unsuccessful, probably because there was no substituent on the N atom of the tetrahydroisoquinoline [44]. If tetrahydroisoquinoline could carry a bulky substituent on the N atom, such as a COOEt group, the corresponding aporphine could be prepared in good yield. Therefore, we ran the acylation of (**II**) with one equivalent ClCOOCH_3_ in CHCl_3_ on ice-bath for 1h to obtain the tetrahydroisoquinoline (**III**) in 88% yield. Radical cyclization of (**III**) using tricyclohexyl phosphine and Pd(OAc)_2_ in dry DMA at 135 °C under N_2_ protection for 5 h then gave intermediate (**IV**) in 84% yield [45]. Afterwards, (**IV**) was deprotected with LiAlH_4_ in anhydrous THF to give nuciferine (**V**) in 56% yield [45].

Previous reports showed that compared with other oxidants including PhI(OAc)_2_, lead(IV) acetate, HIO_4_, and iodobenzene diacetate (IBD), manganese(**III**) acetate was less toxic and could give good yield and less by-products [11]. Therefore, manganese(**III**) acetate was chosen as the oxidizing agent to transform compound (**V**) to lysicamine (LY). The oxidation reaction proceeded in glacial acetic acid at 80 °C for 12 h. Further purification by silica gel chromatography (CH_2_Cl_2_/MeOH/NH_3_(aq) = 98:1:1) retrieved more lysicamine (LY) as yellow needles in 23% yield.

Note that although the final oxidation had very poor yield, the synthesis of compounds (**I**)–(**V**) did not require purification by silica gel chromatography. Instead, crude materials were always used in the next step, and good yields were still obtained (82%–88%, 56% for (**V**)). The overall synthesis required only six steps, whereas the latest report that used Fremy's salt as an effective oxidizing agent required nine steps to prepare the lysicamine end product [16]. Our route is clearly beneficial to the synthesis of a large number of lysicamine and related oxoaporphine alkaloids. We also alleviated the consumption of large amounts of organic solvents that were required in other reports for purification [14, 15].

### Synthesis and structural characterization of 1–4

The metal complexes [Ru(LY)Cl_2_(DMSO)_2_]·3H_2_O(**1**), [Rh(LY-OH)Cl_3_CH_3_OH] (**2**), [Mn(LY)_3_](ClO_4_)_2_·3CHCl_3_ (**3**), and [Zn(LY)_2_(ClO_4_)_2_] (**4**) were synthesized by reaction of LY with cis-RuCl_2_(DMSO)_4_, RhCl_3_·H_2_O, Zn(ClO_4_)_2_·6H_2_O, and Mn(ClO_4_)_2_·6H_2_O in 2:1 MeOH/CHCl_3_, respectively. The metal complexes were characterized by IR, ESI-MS and elemental analyses (^1^H NMR and ^13^C NMR were used for **2**). Their crystal structures were determined by single-crystal X-ray diffraction analysis.

### Crystal structures

**Figure 2:**
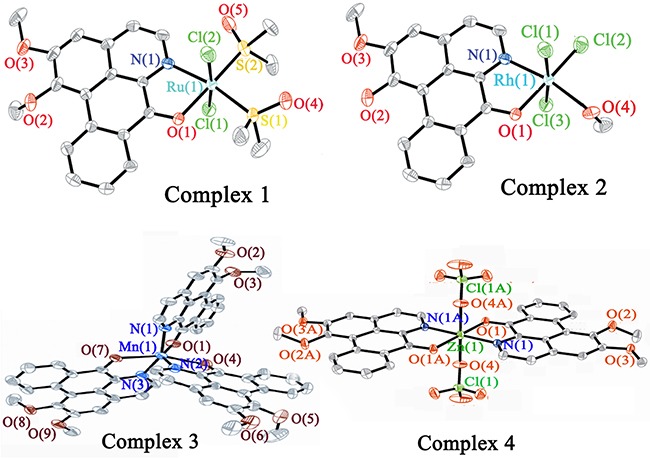
Crystal structures of complexes 1–4

Supplementary Tables 1 and 3 summarize the crystal data and refinement details of **I**, **III**, **IV**, LY and of **1–4**, respectively. Supplementary Tables 2 and 4 list the selected bond lengths and angles of the corresponding compounds. Figure 2 shows the crystal structures of **1–4** (see Supplementary Figure 1 for the crystal structures of **I**, **III**, **IV** and LY). The complexes **1–4** had mononuclear structures and the central metal ion adopted the six-coordinated distort geometry. The Ru^II^ or Rh^III^ ions of **1** and **2** were coordinated by N and O from one LY ligand, two Cl and two DMSO via the S atom (for **1**), three Cl and one methanol (for **2**), to form a distorted octahedral geometry. It is worth noting that in complex **2**, one –OCH_3_ group of LY changed to –OH. In **3** and **4**, the Mn^II^ centre ion was coordinated by N and O from three LY ligands, and the Zn^II^ ion was associated with the N and O atoms from two LY ligands and the O atom from two ClO_4_^−^.

**Table 1:** The ^a^IC_50_ values (μM) of LY, 1–4 and cisplatin for the four tumour cells and the HL-7702 normal liver cell

Compound	BEL-7404	HepG2	NCI-H460	T-24	HL-7702
LY	47.52±1.65	23.90±0.41	19.14±0.18	34.50±1.96	29.27±0.72
**1**	49.52±0.53	57.31±3.32	15.76±2.31	20.68±1.83	69.21±1.81
**2**	18.37±0.14	7.56±2.91	10.01±2.39	14.29±1.05	35.08±3.34
**3**	36.24±1.03	14.51±0.69	12.89±3.62	15.42±1.81	55.36±3.65
**4**	29.67±0.96	35.46±3.99	8.17±1.69	16.25±0.93	34.59±2.21
^b^Cisplatin	12.41±0.38	18.51±0.78	18.29±1.02	28.07±1.88	15.67±1.27

^a^IC_50_ values are presented as the mean ± SD from five independent experiments. ^b^Cisplatin was dissolved at a concentration of 1mM in 0.154 M NaCl [46].

To determine the stability of the metal complexes, the TBS solutions (Tris-KCl buffer solution with pH value of 7.35, containing 1% DMSO) of **1–4** were prepared and the UV-Vis spectra were recorded at 0 h, 2 h, 4 h, 8 h, 12 h and 24 h (Supplementary Figure 2). Complexes **2** and **3** were also examined on HPLC (Supplementary Figure 3). No obvious changes were observed in the UV-Vis spectra and HPLC chromatograms. Hence, **1–4** were stable in TBS and DMSO at room temperature for 24 h.

### Inhibition of the proliferation of human cancer cells

The inhibitory effects of LY, **1**–**4** and the corresponding metal salts were tested against BEL-7404, HepG2, NCI-H460, and T-24 cancer cells and the normal liver cell HL-7702 in the MTT assay with cisplatin as the positive control. All cells were incubated with compounds at 20 μM for 48 h. Supplementary Table 5 shows that the inhibitory rates of LY and **1**–**4** varied in 31%–74% against the selected tumor cells, except for **1** and **4** gave 18.83% and 20.39% inhibition against HepG2 cells, respectively. Remarkably, **2** and **3** were highly cytotoxic against HepG2 cells (74.90% and 62.99%) and against NCI-H460 (63.82% and 54.93%). Besides, **2** and **3** were less toxic to normal liver cell HL-7702 (15.1% and 32.9%) than LY(45.5%) and cisplatin (68.9%). Thus, **2** and **3** may be potential safe candidates as an anticancer agent. Table 1 shows that **2** and **3** had much lower IC_50_ values (7–15 μM) than LY and cisplatin against all selected tumor cell lines except BEL-7404. Among them, HepG2 cells showed the highest sensitivity to **2** and **3** with IC_50_ value of 7.56±2.91 and 14.51±0.69 μM, which were approximately 1.6–3.2 fold increased comparing with the free LY ligand. Therefore, the HepG2 cells were selected for studying the antitumor mechanism of **2** and **3**.

### Uptake of metal complexes in HepG2 cells

The cellular uptake and distribution of the complexes would help to elucidate the target site and pathways [47, 48]. The distribution of the metal (Rh and Mn) in the two cellular fractions of the HepG2 cells was investigated by ICP-MS according to the reported method [49] (see in Supplementary Figure 4). The results showed that after exposing the HepG2 cells to **2** (7.0 μM) or **3** (14.0 μM) for 8 h, the amount of Rh or Mn was higher in the mitochondrial fraction than in the nuclear fraction. Therefore, the mitochondria were a potential target site for **2** and **3**.

### Cell cycle distribution

**Figure 3:**
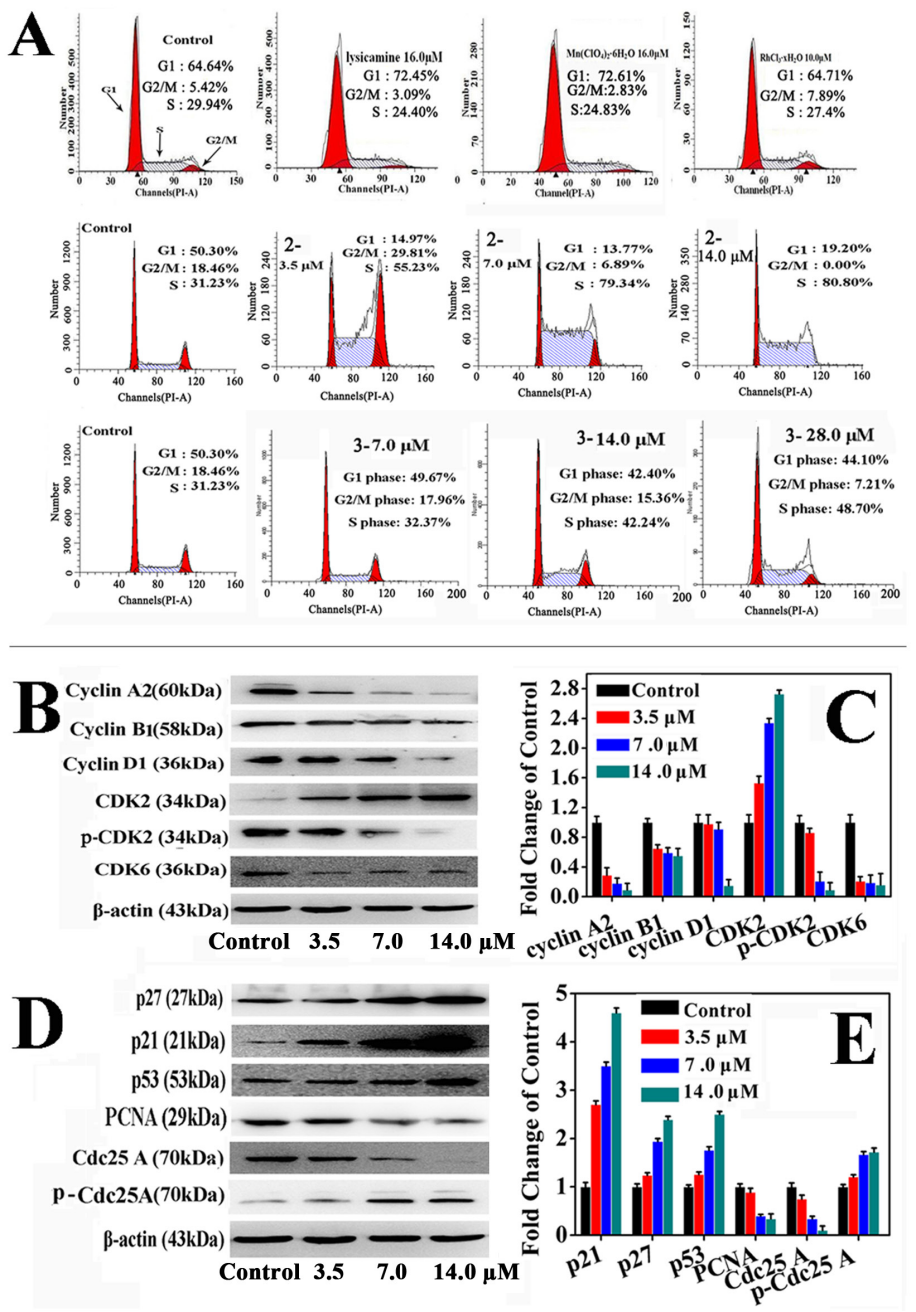
The effects of 2 and 3 treatment in HepG2 cells on cell cycle **(A)** Cell cycle analysis of **2**, **3**, LY and metal salt in HepG2 cells. **(B, D)** Effects of **2** treatment in HepG2 cells on cell cycle regulatory proteins at 3.5, 7.0 and 14.0 μM for 24 h, respectively. **(C, E)** The relative protein expression of each band = (density of each band/density of β-Actin band). Mean ± SD was from three independent measurements.

The effects of **2** and **3** on the cell cycle progression of HepG2 cells were determine by flow cytometry (Figure 3A). Complexes **2** and **3** caused significant cell cycle arrest at the S phase in a dose-dependent manner after incubation for 24 h. In the presence of **2** at 3.5, 7.0, and 14.0 μM, the population of the HepG2 cells in the S phase increased from 31.23% to 55.23%, 79.34% and 80.80%, respectively. Similarly, the population of HepG2 cells in the S phase increased from 31.23% to 48.70% when 28.0 μM **3** was applied. The findings were consistent with the results of the *in vitro* cytotoxicity and also indicated that **2** was more active than **3** towards HepG2 cells. In contrast, Mn(ClO_4_)_2_·6H_2_O (16.0 μM) and RhCl_3_·3H_2_O (16.0 μM) did not significantly induce the cell cycle arrest. In brief, we assumed that the antiproliferative effects of **2** and **3** resulted mainly from inducing cell cycle arrest in the S phase. Since LY only blocked the cell cycle in the G1 phase, the cytotoxicity of **2** and **3** must come from different mechanisms. The unique activities of **2** and **3** must be ascribed to the Rh(III) and Mn(II) center in the complexes.

### Expression of proteins related to cell cycle

HepG2 cells were incubated with **2** and **3** at increasing concentrations for 24 h, and the expression of cell cycle regulatory proteins in the S phase was examined through Western blot analysis. Figure 3B–3E and Supplementary Figure 5 shown that **2** and **3** decreased the expression of cyclin A2, cyclin B1, cyclin D1, cyclin E1, Cdc25A, p-Cdk2, and Cdk6 in a concentration-dependent manner. In contrast, **2** and **3** increased the expression of Cdk2, p-Cdc25A, p53, p21 and p27, also in a concentration-dependent manner. It could thus be concluded that **2** and **3** induced S phase arrest in the cell cycle by activating the p53, p21 and p27 proteins and inhibiting cyclin A2/CDK2, cyclin D1/CDK2,6, and cyclin E1/CDK2 [50–52]. Furthermore, **2** and **3** also decreased the proliferating cell nuclear antigen (PCNA) level, which is a key factor in DNA replication and cell cycle regulation [53], indicating that DNA synthesis was inhibited and the cell cycle was arrested in the S phase [54].

### Cell apoptosis

**Figure 4:**
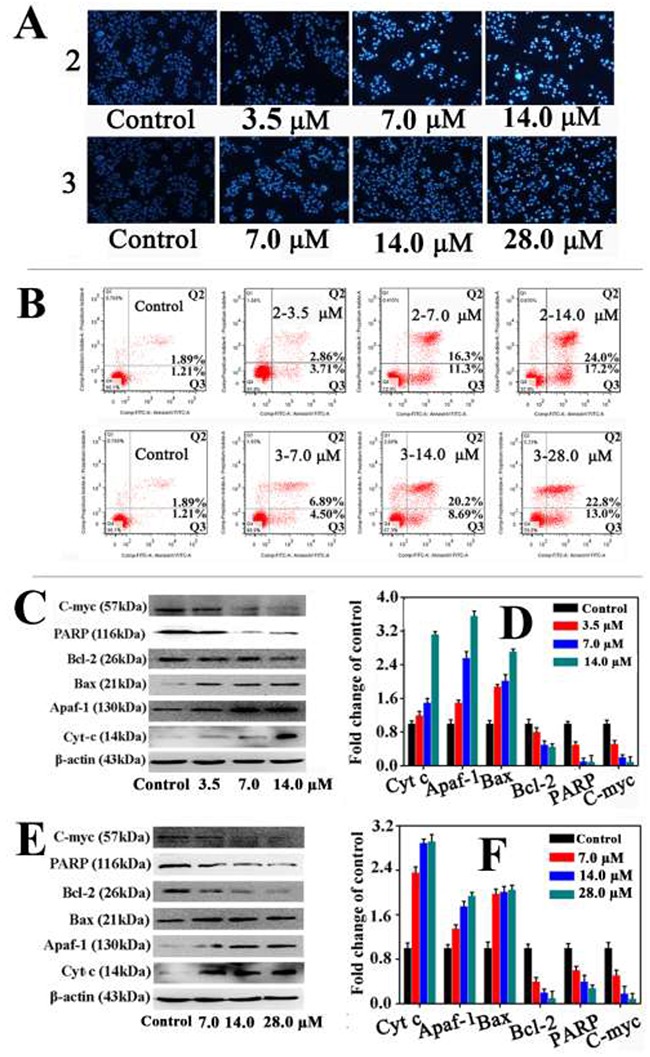
Apoptosis of HepG2 cells induced by 2 and 3 **(A)** The apoptotic nuclear morphological analysis by Hoechst-33258 staining(magnification 100×) and **(B)** the apoptosis of HepG2 cells analysis by flow cytometry after 24 h treatment with **2** and **3** at variously concentrations for 24 h. **(C, E)** Western blot analysis of apoptosis associated proteins after treatment of HepG2 cells with **2** (3.5, 7.0 and 14.0 μM) and **3** (7.0, 14.0 and 28.0 μM) for 24 h, respectively. **(D, F)** Densitometry analysis from part C and E. The relative expression of each band = (density of each band/density of β-actin band). Mean and SD values were from three independent measurements.

The Hoechst33258 assay is commonly used to measure the changes in cell morphology caused by apoptosis [55]. The HepG2 cells were incubated with **2** (3.5, 7.0, and 14.0 μM) and **3** (7.0, 14.0, and 28.0 μM) for 24 h, respectively, then stained with Hoechst33258 and examined under a fluorescence microscope (Figure 4A). Compared with control HepG2 cells, these cells treated with **2** and **3** gave stronger fluorescence in a dose-dependent manner, and the changes in cell morphology included irregular nuclei, chromatin shrinkage, and formation of apoptotic bodies, all of which indicated cell apoptosis.

To further examine the apoptosis of HepG2 cells triggered by **2** and **3**, the cells were incubated with **2** and **3** for 24 h, then stained with both Annexin V-FITC and PI before analysis by flow cytometry [56]. Figure 4B shows that treatment with **2** (3.5, 7.0, and 14.0 μM) and **3** (7.0, 14.0 and 28.0 μM) significantly increased the percentage of apoptotic cells (Q2 and Q3). After treating the HepG2 cells with 14.0 μM **2** (respectively 28.0 μM **3**), the percentages of cells undergoing apoptosis and necrosis increased from 4.1% to 41.2 % (respectively to 35.8 %).

### Expression of apoptosis-related proteins

To further confirm that **2** and **3** induced apoptosis in HepG2 cells, Western blot analysis was used to examine the protein expression of C-myc, Bcl-2, Bcl-xl, Bax, Apaf-1 and cytochrome *c* (Figure 4C-4F).

After the HepG2 cells were incubated with **2** and **3** for 24 h at various concentrations, the expression of C-myc, Bcl-xl and Bcl-2 decreased, and the expression of Bax, Apaf-1 and cytochrome *c* increased. The degree of increase (or decrease) depended on the concentration of **2** and **3**. It could be concluded that the increase in pro-apoptotic factor (Bax) formed the mitochondrial apoptosis-induced channel (MAC), which mediated the release of cytochrome *c* from mitochondria to the cytosol, and promoted the activation of caspase-9/-3, ultimately leading to cell apoptosis [57].

### The level of reactive oxygen species (ROS), intracellular Ca^2+^, and mitochondria membrane potential (ΔΨm) in HepG2 cells

The cell uptake assay showed that the amount of Rh and Mn was higher in the mitochondria than in the nuclear fraction, and indicated that **2** and **3** probably targeted the mitochondria to cause cell apoptosis. The overproduction of ROS [58, 59], increase of intracellular Ca^2+^ [60], and loss of mitochondrial membrane potential (MMP, ΔΨm) [61] can all lead to mitochondrial dysfunction, and they thus play a central role in apoptosis [62].

**Figure 5:**
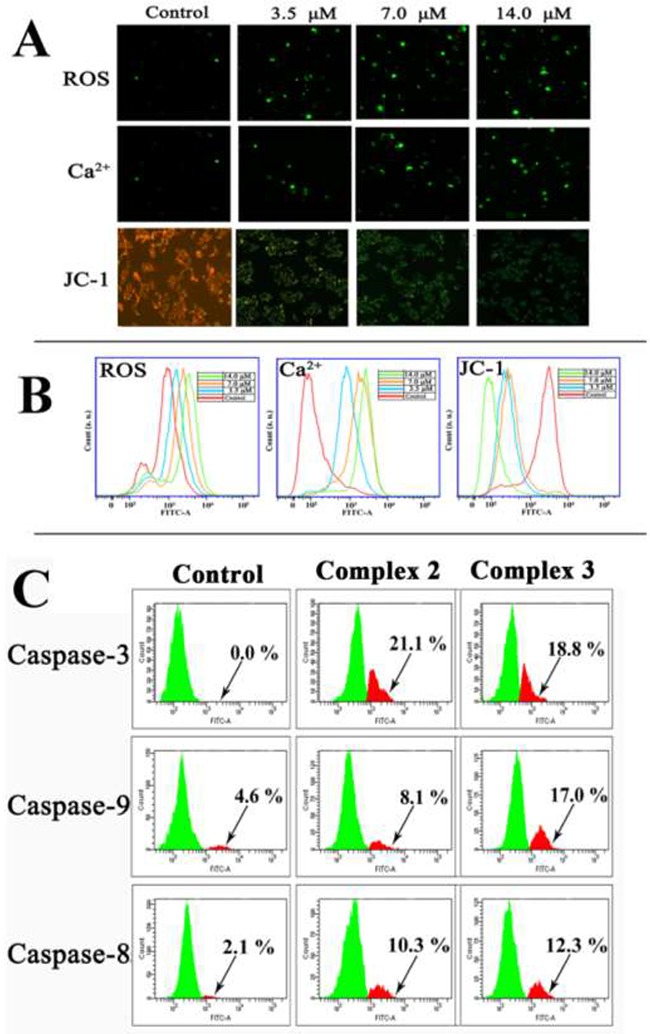
The effect of 2 or 3 on the levels of ROS, intracellular Ca^2+^, loss of ΔΨm and the activated caspase-3/8/9 expression after HepG2 cells were treated with 2 and 3 for 24 h, respectively **(A)** The images of fluorescence microscope (magnification 100×). **(B)** The change of ROS, Ca^2+^ and ΔΨm examined by flow cytometry assay. **(C)** Flow cytometry analysis of activated caspase-3/8/9 expression in HepG2 cells after incubated with **2** and **3** at IC_50_ values for 24 h.

The effects of **2** and **3** on the ROS, intracellular Ca^2+^ and ΔΨm of HepG2 cells are shown in Figure 5 and Supplementary Figure 6. The changes in the ROS level, intracellular Ca^2+^ and loss of ΔΨm in the HepG2 cells were detected by the DCFH-DA, Fluo-3 AM, and JC-1 fluorescent probes, respectively (see in Figure 5A). Green fluorescence represented higher ROS levels, more intracellular Ca^2+^, and reduced ΔΨm. In the control cells, little green fluorescence was observed, suggesting that the levels of ROS and Ca^2+^ were very low. The cells exposed to **2** (3.5, 7.0 and 14.0 μM) for 24 h gave green fluorescence with escalating intensity in a dose-dependent manner, which indicated the increase of ROS and Ca^2+^, as well as the loss of ΔΨm. As a confirmation, flow cytometry analysis (Figure 5B) also showed that **2** greatly increased the ROS and Ca^2+^ levels and promoted the loss of ΔΨm in the HepG2 cells (blue, orange, and green lines). Compared with the control cells (red line), the treated cells differed notably in the intracellular Ca^2+^level and the loss of ΔΨm. Similarly, treating the HepG2 cells with **3** (7.0, 14.0 and 28.0 μM) for 24 h also caused overproduction of ROS, rising intracellular Ca^2+^, and loss of Δψm (Supplementary Figure 6). Therefore, it is highly possible that **2** and **3** induced cell apoptosis via the mitochondrial pathway.

### Activation of caspases-3/-8/-9 in HepG2 cells

Caspases are considered to be the central executioners of cell apoptosis. The activation of caspase-3/-9 is an essential factor in the mitochondrial apoptotic pathway [63]. The activation of casepase-3 and casepase-9 in HepG2 cells caused by **2** and **3** were analysed by flow cytometry after the cells were treated **2** and **3** at IC_50_ for 24 h. The proportion of activated caspase-3, -9 and -8 cells after treatment with complex **2** were 21.1%, 8.1%, and 10.3% of total cells, respectively (Figure 5C). Similarly, complex **3** increased the activation of caspase-3, -9 and -8 were 18.8%, 17.0% and 12.3%, respectively. These results suggested that **2** and **3** were efficient activators of caspase-3, -9 and -8 and induced apoptosis in HepG2 cells via the caspase-dependent mitochondrial pathway.

### Expression of genomes related to cell cycle and apoptosis

**Figure 6:**
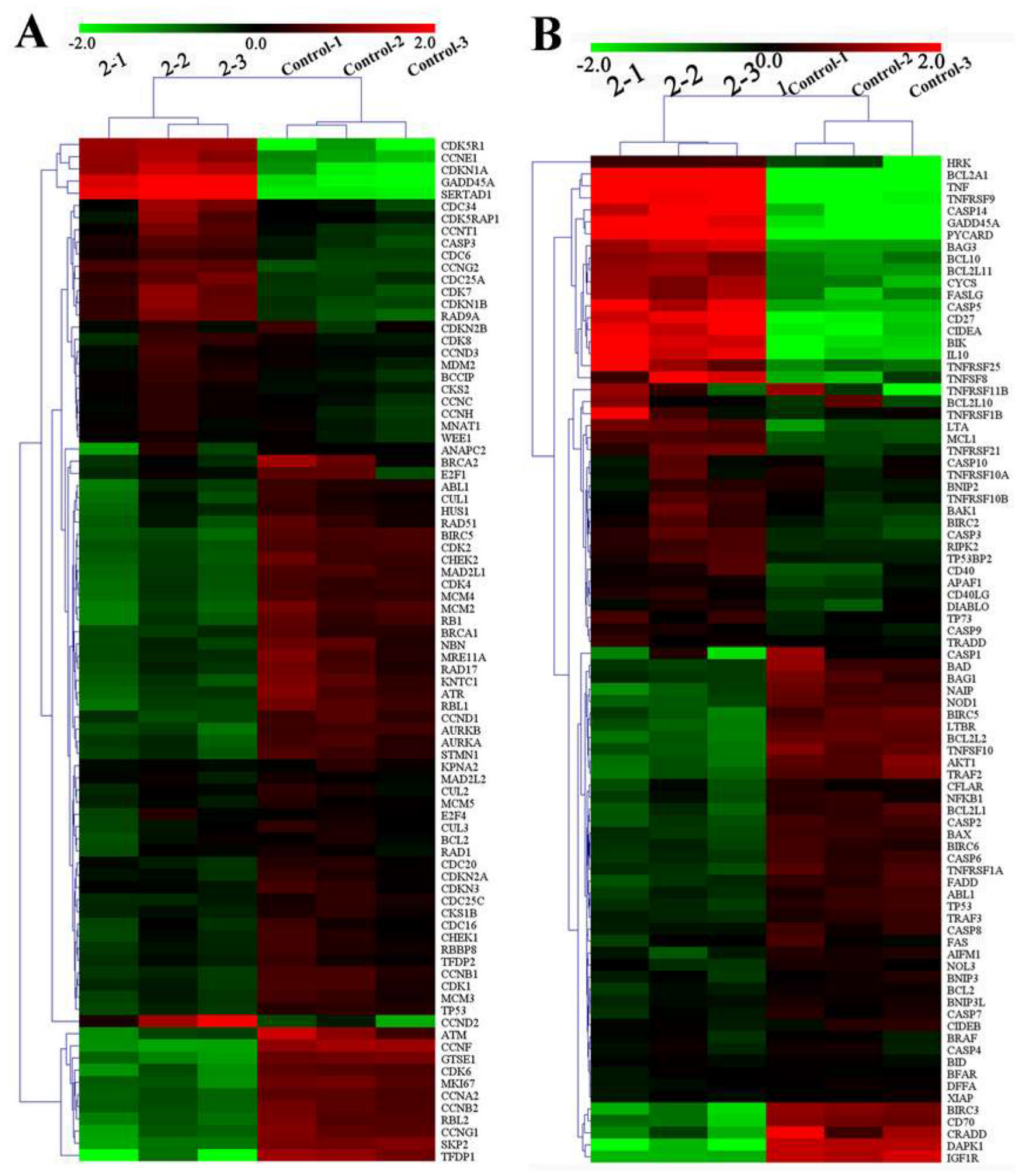
Relative expression profiles of 89 genes in HepG2 cells after being treated with 2 (7.0 μM) for 24 h **(A)** Cell cycle related genes and **(B)** apoptosis-related genes.

Gene chip is widely used in screening antitumor drugs and pharmacological studies because of its high sensitivity, high flux, miniaturization, and automation [64–66]. Here we examined the expression of mRNA genes that were relevant to cell cycle and apoptosis in the HepG2 cells. Specifically, the HepG2 cells were treated with **2** (7.0 μM) and **3** (14.0 μM) for 24 h and then analyzed by RT-qPCR array. As shown in Figure 6A, Supplementary Figure 7, Supplementary Tables 6 and 7, of the 89 cell cycle regulators genes, 58 and 31 genes were differentially expressed in mRNA levels by 1.5-fold or more after treated with **2** (7.0 μM) and **3** (14.0μM) for 24 h, respectively. The decreased genes included CCNA2 (Cyclin A2), CCNB1 (Cyclin B1), CCND1(Cyclin D1), CDK2, CDK6, and CHEK2 (Chk2). The activated genes included CDKN1A (p21^Cip1^) and CDKN1B (p27^KiP1^), agreed well with the protein expression results from Western blot analysis. It could be concluded that **2** and **3** arrested the cell cycle in the S phase by altering the expression of related genes and proteins, such as the checkpoint protein and the cyclin–Cdk complexes.

Furthermore, of the 89 apoptosis-related genes, 61 and 34 genes were differentially expressed in mRNA levels by 1.5-fold or more after treated with **2** (7.0 μM) and **3** (14.0 μM) for 24 h, respectively (Figure 6B, Supplementary Figure 8, Supplementary Tables 8 and 9). For **2**, the activated mRNA genes included CASP3, CASP9, CYCS (cytochrome *c*), and APAF1, and BCL2 was decreased. The results here again agreed well with the protein expression results from Western blot analysis. In addition, the expression levels of some mRNA genes changed significantly, e.g., TNF up to 70. 12-fold, BCL2A1 up to 63.84-fold, CASP14, GADD45A, HRK, PYCARD, and TNFRSF9 up to 20.12–33.25-fold, BIK, CD27, CIDEA, and IL10 up to 12.41–13.56-fold, and DAPK1 down to 10.41-fold. Complex **3** had similar effects on the apoptosis genes, but the changes were less pronounced than in the case of **2**.

It is worth noting that tremendous changes occurred in the expression levels of death receptor genes including TNF, TNFRSF1A/1B (TNFR1/R2), TNFRSF 8, 9, 10, 21, 25, FAS, FADD and FASLG. As mentioned above, the initiator caspase-8 and the executioner caspase-3 (the downstream protein of death receptor) were activated after the cells were incubated with **2** and **3**. Therefore, it can be inferred that **2** and **3** promoted the apoptosis of tumour cells not only through the mitochondria-mediated intrinsic pathway but also through the death receptor-mediated extrinsic pathway [67].

### DNA binding studies

Many findings show that metal complexes can induce cell apoptosis by DNA damage mediated S phase cycle cell arrest [68, 69]. Since **2** and **3** caused the S phase arrest in HepG2, we found it worthy to evaluate the binding ability of **2** and **3** to DNA.

**Figure 7:**
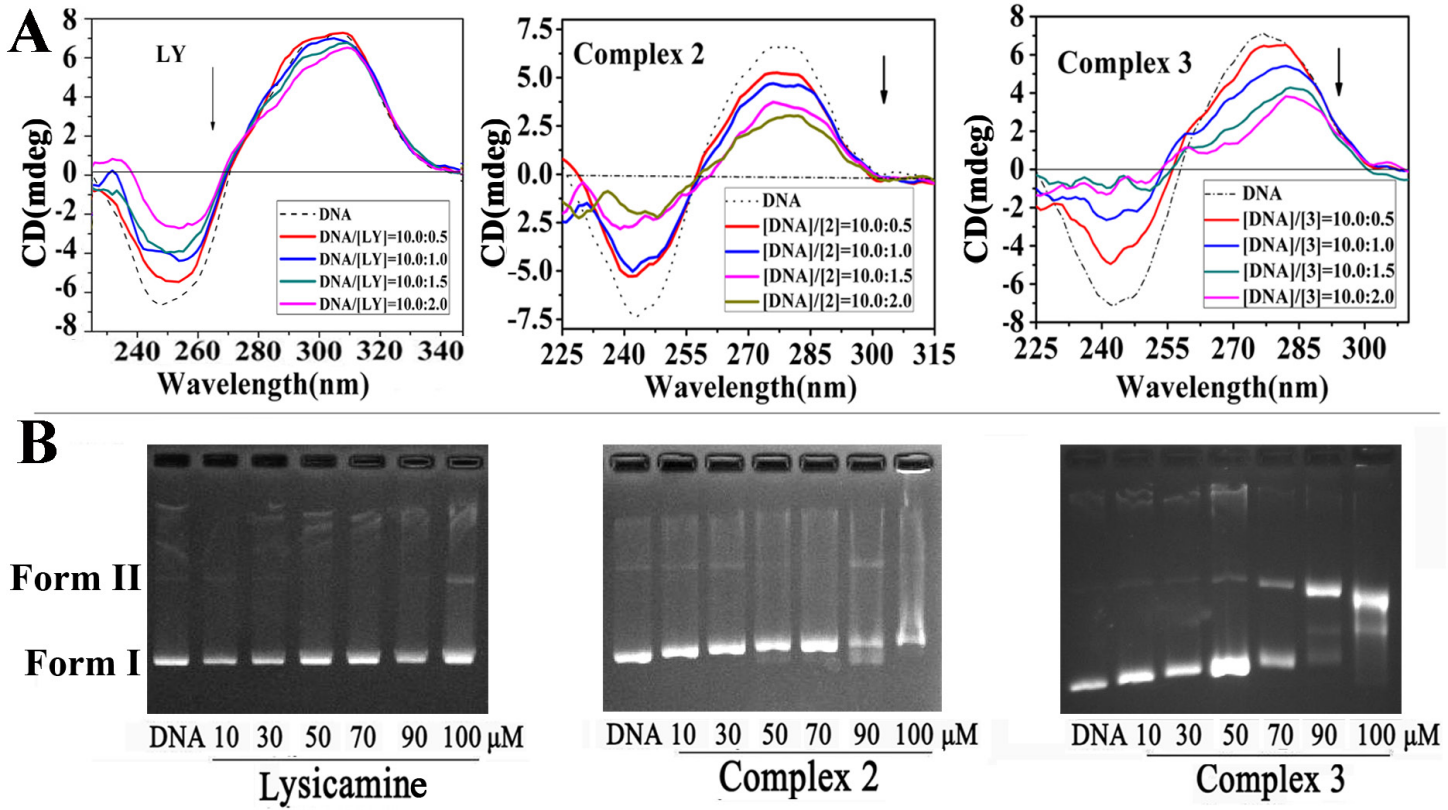
**(A)** Circular dichroism spectra of ct-DNA bound to LY, **2** and **3** with [DNA]/[each compound] ratios range were 10:0. 5, 10:1.0, 10:1.5 and 10:2.0 (the concentration of ct-DNA bases alone of 1×10^−4^ M, dashed line). **(B)** Agarose gel electrophoresis mobility shift assay of pBR322 plasmid DNA (0.5 μg/μL) when interacted with LY, **2** and **3** with increasing concentrations from 10 to 100 μM.

Circular dichroism (CD) is highly sensitive to the changes in the secondary structure of nucleic acids [70]. Covalent or intercalative binding may change the CD spectra of DNA by altering the tertiary structure of DNA [71]. We determined the CD spectra of ct-DNA in the presence of **2**, **3** and LY, respectively (Figure 7A). The addition of LY decreased in negative absorption evidently but changed the positive absorption only slightly. Therefore, intercalation was the most probable binding mode between LY and DNA since LY had a planar structure, and the binding was weak. In contrast, the addition of **2** or **3** caused significant hypochromism in both the negative and the positive bands of the CD spectrum, and the hypochromism intensified with rising [complex]/[DNA] ratio. The results indicated that the DNA formed a stable combination with **2** or **3** [72], and the helicity and base stacking of the DNA were altered [73]. Obviously, compared with LY, **2** and **3** demonstrated stronger intercalative ability of to DNA, which could be attributed to the coordination of metal ions.

The DNA cleavage activity of **2**, **3** and LY were tested by agarose gel electrophoresis assay using pBR322 plasmid DNA (0.5 μg/μL) in TBE buffer (pH=8.5). Figure 7B shows that the addition of LY did not change the supercoiled DNA and its migration rate. In contrast, under the same experimental conditions, when the concentration of **2** increased from 10 μM to 50 μM, the mobility of supercoiled DNA (form I) was reduced, but the relaxed form (form II) was not observed. When the concentration of **2** increased further to 70 μM, the form II DNA appeared. Almost all supercoiled DNA (form I) changed to relaxed DNA (form II) when the concentration of **2** reached 90 and 100 μM. It could be concluded that **2** could cleave supercoiled DNA, and the coordinated metal probably played a key role in such cleavage [74]. In contrast, the cleavage of supercoiled DNA was still little even when the concentration of **3** reached 90 and 100 μM. Hence, compared with **3**, **2** had stronger binding ability to the plasmid DNA.

### *In vivo* safety profile

The safety profile of **2** was test *in vivo* on male and female KM mice. A single intraperitoneal injection of high concentration of **2** (0.57 mg/mL in 10% DMSO) was made to KM mince at 0.4 mL/10 g (*i.e*., 22.8 mg/kg). On the next day, some symptoms were observed, including slouching, slow movement, hair loss, anorexia, and the body weight decreased significantly in all treatment groups (15% body weight loss). There was no death of mice during the observation period (14 days), and 22.8 mg/kg was thus considered as the maximum tolerable dose (MTD) [75, 76]. However, the administration of **2** at MTD every three days led to much more serious the symptoms in the mice, including loose stools and lost their body weight significantly in these assay. Further study revealed that a single intraperitoneal injection of **2** at a reduced dose of 1/3 MTD (7.8 mg/kg) caused the KM mice to lose only <10% body weight on the next day, and the weight loss was fully recovered on day 3. When **2** was given at 1/3 MTD on every two days for 14 days, there was no animal death and the body weight of the mice grew slowly, suggesting that the dose at 1/3 MTD (i.e., 7.6 mg/kg) was safe to KM mice and could be used *in vivo* xenograft studies.

### *In vivo* HepG2 xenograft study

**Figure 8:**
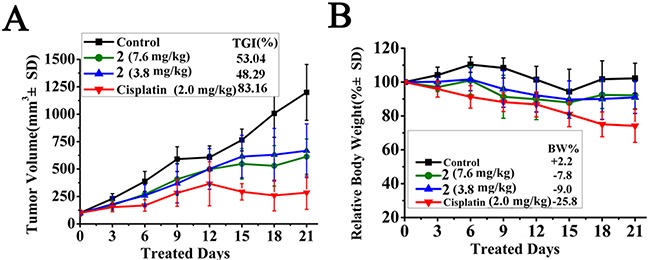
*In vivo* anticancer activity of 2 in HepG2 xenograft model **(A)** Tumour volume vs days of treatment with **2** (7.6, 3.8 mg/kg/2 days), cisplatin (2.0 mg/kg/2days), or vehicle. Tumour growth is tracked by the mean tumour volume (mm^3^) ± SD (n=6) and calculated as tumour growth rate [TGI%] values. **(B)** Body weight change. Relative body weight by considering the body weight at the start of the treatment as 100%, the percent weight loss or gain was calculated on subsequent days of treatment.

The HepG2 xenograft model was further used to evaluate antitumor activity of **2** *in vivo*. As illustrated in Figure 8, **2** showed could inhibit tumour growth at 1/3 and 1/6 MTD (7.6 and 3.8 mg/kg per 2 days, respectively), and the TGI [77] was approximately 53.04% and 48.29%, respectively (p < 0.01). There was no obvious dose-dependent relationship. Although cisplatin could inhibit tumour growth more effectively than **2**in the HepG2 xenograft model (TGI = 83.16%, p<0.001), the administration of cisplatin caused a sharp loss in body weight (25.8%) that exceeded the permissible value (*i.e*., 20%) [77]. In contrast, the body weight loss of the mice was much less (<10%) when they were treated with **2**. These results indicated that **2** had good antitumor activity *in vivo* when administered at 1/3 MTD and was safer than cisplatin.

## MATERIALS AND METHODS

### Synthesis and characterization of LY and complexes 1–4

#### Synthesis of compound (I)

2-Bromophenylacetic acid (0.2 mol) was dissolved in 200 mL CHCl_3_, then 70 mL SOCl_2_ was added by slowly dropping, reflux at 75°C for 2 h, the intermediate product was obtained after evaporate the solvent. Then, intermediate product dissolved in chloroform was slowly added to 0.2 mol 3,4-dimethoxyphenethylamine CHCl_3_ solution contain 300 mL saturated NaHCO_3_. The mixture reacted on ice for 2 h, the chloroform layer washed three times by water, the white needle crystal of (**I**) were obtained recrystallized from methanol, yield of 80%. ESI-MS m/z: 379.2 [M+H]^+^; ^1^H-NMR (500 MHz, d_6_-DMSO): *δ* 8.08 (t, *J* = 5.6 Hz, 1H), 7.60–7.56 (m, 1H), 7.33–7.28 (m, 2H), 7.20–7.16 (m, *J* = 8.0, 6.0, 3.1 Hz, 1H), 6.84 (dd, *J* = 14.8, 5.1 Hz, 2H), 6.73 (dd, *J* = 8.1, 2.0 Hz, 1H), 3.74 (s, 3H, OCH_3_), 3.73 (s, 3H, OCH_3_), 3.60 (s, 2H), 3.33 (dd, *J* = 13.2, 7.0 Hz, 2H), 2.70 (t, *J* = 7.3 Hz, 2H). ^13^C-NMR (125 MHz, *d*_6_-DMSO): *δ* 169.21, 149.11, 147.72, 136.49, 132.69, 132.38, 132.23, 129.00, 127.97, 124.94, 120.99, 113.03, 112.37, 55.99, 55.84, 42.85, 41.03, 35.17. Elemental analysis, calcd (%) for C_18_H_2_0BrNO_3_: C 57.15, H 5.33, N 3.70, O 12.69; found: C 57.27, H 5.24, N 3.68, O 12.74. (Spectra of ESI-MS and ^1^H/^13^C NMR See in Supplementary Figures 9 and 10).

#### Synthesis of compound (II)

Compound (**I**) (0.2 mol) and POCl_3_ (60 mL) in dry toluene (300 mL) was refluxed at 85 °C for 3 h, after removal of the organic solvent, the residue dissolved in CHCl_3_ and adjust the pH of the solution to about 9 with ammonia. The organic layer washed with saturated NaHCO_3_ solution (200 mL×2) followed by water (200 mL×2), and concentrated under reduced pressure. The resulting residue was used for the next step without further purification. The residue was dissolved in CH_3_OH and CH_2_Cl_2_ system (v/v,1:1) and added 0.2 mol sodium triacetoxyborohydride (STAB), reacted at room temperature for 1 h, then evaporated. The residue was dissolved in CH_2_Cl_2_ and then wash with saturated NaHCO_3_ solution (200 mL×2) followed by water (200 mL×2). The residue was purified through recrystallized from ethanol to afford compound (II), yield of 82 %. ESI-MS m/z: 362.3 [M+H]^+^; ^1^H NMR (500 MHz, CDCl_3_): *δ* 7.56 (d, *J* = 7.8 Hz, 1H), 7.26–7.23 (m, 2H), 7.14–7.10 (m, 1H), 6.58 (s, 1H), 6.27 (s, 1H), 4.50 (t, *J* = 7.1 Hz, 1H), 3.83 (s, 3H, OCH_3_), 3.62 (s, 3H, OCH_3_), 3.41–3.17 (m, 4H), 2.93–2.86 (m, 2H), 1.86 (s, 1H, NH); ^13^CNMR (125 MHz, CDCl_3_): *δ* 176.82, 148.08, 147.08, 137.08, 133.05, 132.35, 128.66, 127.66, 126.56, 125.20, 111.42, 109.78, 55.81, 55.64, 54.06, 41.73, 38.77, 27.04; Elemental analysis, calcd (%) for C_18_H_2_0BrNO_2_: C 59.68, H 5.56, N 3.87, O 8.83; found: C 59.61, H 5.47, N 3.92, O 8.84. (Spectra of ESI-MS and ^1^H /^13^C NMR See in Supplementary Figures 11 and 12).

#### Synthesis of compound (III)

The 0.2 mol compound (**II**) dissolved in 150 mL CHCl_3_ and added NaOH solution (0.2mol/L), stirring, then 0.2 molClCOOCH_3_ (dissolved in CHCl_3_) was slowly added. The mixture keep stirring on ice for 1 h, then the organic layer washed with water two times, and concentrated under reduced pressure. The residue recrystallized in methanol, and the white needle crystal (**III**) were obtained, yield of 88%. ESI-MS m/z: 419.3 [M-H]^-^; ^1^H NMR (500 MHz, *d_6_*-DMSO): *δ* 7.69–7.65 (m, 1H), 7.28–7.22 (m, 1H), 7.18–7.15 (m, 1H), 7.00 (s, 1H), 6.82 (d, *J* = 5.8 Hz, 1H), 6.78 (s, 1H), 3.84 (s, 3H, OCH_3_), 3.78 (s, 3H, OCH_3_), 3.74 (s, 3H, OCH_3_), 3.72–3.70 (m, 3H), 3.15–3.09 (m, 4H), 2.83 (d, *J* = 6.3 Hz, 2H). ^13^C NMR (125 MHz, *d_6_*-DMSO): *δ* 155.08, 149.97, 148.08, 146.46, 133.01, 132.68, 130.09, 129.54, 129.29, 128.88, 128.3, 112.54, 107.30, 56.25, 55.97, 55.85, 55.03, 53.21, 44.55, 43.81, 28.47. Elemental analysis, calcd (%) for C_20_H_22_BrNO_4_: C 57.15, H 7.28, N 3.33, O 15.23; found: C 57.21, H 7.21, N 3.40, O 15.30. (Spectra of ESI-MS and ^1^H /^13^C NMR See in Supplementary Figures 13 and 14).

#### Synthesis of compound (IV)

10.0 g compound (**III**), 1.4 g tricyclohexyl phosphine, 11.5 g K_2_CO_3_ and 0.53 g Pd(Ac)_2_ were dissolved in dry DMA, the suspension was heated to 135 °C under N_2_ for 5 h. After the completion of the reaction (monitored by TLC), DMA was distilled off under high vacuum, and the residue was dissolved in chloroform (200 mL) and washed with saturated NaHCO_3_ solution (100 mL×2), followed by water (100 mL×2). The organic layer was concentrated under reduced pressure and purified though recrystallized in methanol, the white needle crystal (compound IV) were obtained, yield of 84%. ESI-MS m/z: 339.4 [M+H]^+^;^1^H NMR (500 MHz, CDCl_3_): δ 8.46 (d, *J* = 7.9 Hz, 1H), 7.37–7.33 (m, 1H), 7.28 (d, *J* = 3.4 Hz, 2H), 6.70 (s, 1H), 3.92 (s, 3H, OCH_3_), 3.78 (s, 3H, OCH_3_), 3.68 (s, 3H, OCH_3_), 3.28 -3.20 (m, 1H), 3.03–2.84 (m, 4H), 2.02–1.89 (m, 2H); ^13^CNMR (125 MHz, CDCl_3_): δ 152.08, 150.96, 145.65, 136.77, 131.70, 128.49, 128.38, 127.68, 127.04, 126.68, 126.13, 125.87, 111.48, 60.04, 56.47, 55.95, 43.33, 38.93, 30.21, 27.64; Elemental analysis, calcd (%) for C_20_H_21_NO_4_: C, 70.78; H, 6.24; N, 4.13; O, 18.86; found: C, 70.83; H, 6.21; N, 4.20; O, 18.21. (Spectra of ESI-MS and ^1^H /^13^C NMR See in Supplementary Figures 15 and 16).

#### Synthesis of compound (V)

8.8g compound (IV) and 8.0 g LiAlH_4_ were dissolved in dry THF (600 mL) under N_2_ on ice, then the suspension heated to 65°C for 5 h. After removal of THF, the residue was dissolved in ethyl acetate, and ammonia was added until no bubbles on the ice bath. The organic layer was concentrated under reduced pressure and purified though recrystallized in methanol, the blackish green needle crystal (compound V) were obtained, yield of 56%. ESI-MS m/z: 296.3[M+H]^+^;^1^H NMR (500 MHz, CDCl_3_): *δ* 8.40 (d, *J* = 7.8 Hz, 1H), 7.34 (t, *J*= 7.1 Hz, 1H), 7.30–7.24 (m, 2H), 6.66 (s, 1H), 3.91 (s, 3H, OCH_3_), 3.68 (s, 3H, OCH_3_), 3.20–3.06 (m, 4H), 2.74–2.65 (m, 2H), 2.58 (s, 3H, CH_3_), 2.55–2.52 (m, 1H); ^13^C NMR (125 MHz, CDCl_3_): *δ* 152.04, 145.18, 136.41, 132.14, 128.66, 128.34, 127.89, 127.83, 127.36, 1127.03, 126.90, 111.29, 62.34, 60.25, 55.87, 53.28, 43.93, 35.08, 29.16; Elemental analysis, calcd (%) for C_19_H_21_NO_2_: C 77.26, H 7.17, N 4.74, O 10.83; found: C 77.28, H 7.11, N 4.76, O 10.89. (See in Supplementary Figures 17 and 18).

#### Synthesis of lysicamine (LY)

0.02 mol compound (**V**) and 0.7 mol Mn(Ac)_3_ were dissolved in 500 mL glacial acetic acid, 80 °C reflux for 12 h(monitored by TLC). Glacial acetic acid was distilled off under high vacuum, and the residue was purified though recrystallized from methanol, purification by silica gel chromatography (CH_2_Cl_2_/MeOH/NH_3_(aq) = 98:1:1), the blackish green crystal (Lysicamine) were obtained, yield of 23%. ESI-MS m/z: 292.1 [M+H]^+^; ^1^H NMR (500 MHz, *d_6_*-DMSO): δ 9.14 (d, *J* = 8.3 Hz, 1H), 8.86 (d, *J* = 5.3 Hz, 1H), 8.38 (d, *J* = 7.7 Hz, 1H), 8.17 (d, *J* = 5.3 Hz, 1H), 7.91 (t, J = 7.8 Hz, 1H), 7.76 (s, 1H), 7.68 (t, J = 7.5 Hz, 1H), 4.10 (s, 3H), 4.02 (s, 3H); ^13^C NMR (125 MHz, *d_6_*-DMSO): *δ* 182.03, 156.96, 152.57, 145.08, 135.71, 135.13, 134.37, 131.99, 129.41, 129.10, 128.71, 128.45, 124.71, 121.93, 118.61, 108.30, 60.93, 56.90; Elemental analysis, calcd (%) for C_18_H_13_NO_3_: C 74.22, H 4.50, N 4.81, O 16.48; found: C 74.25, H 4.46, N 4.77, O 16.53. (Spectra of ESI-MS and ^1^H /^13^C NMR See in Supplementary Figures 19 and 20).

### Synthesis of 1–4

#### Synthesis of [Ru(LY)Cl_2_(DMSO)_2_]·3H_2_O (1)

Cis-RuCl_2_(DMSO)_4_ was repaired according the report [78], 1.0 mmol LY (0.0146 g) and 0.1 mmol cis-RuCl_2_(DMSO)_4_ (0.049 g) were dissolved in CHCl_3_CH_3_OH solution (v/v,1:1), reflux for 12 h in 65 °C water bath, then cooling and filter, the filtrated solution was allowed to evaporate slowly for a week, green block crystals suitable for X-ray singe-crystal diffraction analysis were harvested, yield of 50%. IR(KBr): (-NH) 3452 (m), (Ar-H) 3008 (m), (C=O) 1601 (m), (C=C) 1563, 1481, 1468 (s), (C-O) 1412, 1269 (vs), (C-N) 1026 (s) cm^-1^; ESI-MS m/z: 619 [M+H]^+^, 652 [M+CH_3_OH+H]^+^; Elemental analysis, calcd (%) for C_22_H_31_Cl_2_NO_8_RuS_2_: C, 42.88; H, 3.755; N, 2.29; Found. C, 42.65; H, 4.07; N, 2.26. (Spectra of ESI-MS See in Supplementary Figure 21).

#### Synthesis of [Rh(LY-OH)Cl_3_CH_3_OH]·H_2_O (2)

LY (0.1 mmol, 0.0146g), 0.1 mmol RhCl_3_·3H_2_O (0.0209 g), methanol (1.0 mL), chloroform (1.0 mL) were placed in a thick Pyrex tube (ca 20 cm long), the mixture was frozen by liquid N_2_, and then evacuated under vacuum and sealed. Then it was heated at 80 °C for 72 h. Then the dark red bulk crystals suitable for single-crystal X-ray diffraction analysis were harvested, about yield of 56%. IR(KBr): (-NH) 3357 (m), (Ar-H) 3066 (m), (C=O) 1602 (m), (C=C) 1557, 1496, 1429 (s), (C-O)1278 1214(vs), (C-N) 1077 (s) cm^-1^; ESI-MS m/z: 516.81, [M-H]^-^; Elemental analysis, calcd (%) for C_18_H_15_Cl_3_NO_5_Rh: C, 40.58; H, 2.724; N, 2.81; Found. C, 41.86; H, 2.735; N, 2.72; ^1^H NMR (500 MHz, *d_6_*-DMSO): *δ* 10.02 (d, *J* = 8.7 Hz, 1H), 9.24 (d, *J* = 5.3 Hz, 1H), 8.54 (d, *J* = 7.3 Hz, 1H), 8.22 (d, *J* = 5.4 Hz, 1H), 7.91 (s, 1H), 7.59 (s, 1H), 7.37 (s, 1H), 4.01 (s, 3H). ^13^C NMR (125 MHz, *d_6_*-DMSO): *δ* 181.52, 173.11, 159.32, 143.94, 138.45, 136.69, 136.65, 134.38, 127.14, 126.18, 125.43, 124.81, 124.70, 123.94, 107.74, 105.41, 56.52, 49.08. (Spectra of ESI-MS and ^1^H /^13^C NMR See in Supplementary Figures 22-24).

#### Synthesis of [Mn(LY)_3_](ClO_4_)_2_·3(CHCl_3_)(3)

The same method was employed by **2**, yield of 68%. IR(KBr): (-NH) 3424(m), (Ar-H) 2942(m), (C=O) 1609(m), (C=C) 1588, 1480, 1466(s), (C-O) 1412, 1269(vs), (C-N) 1096(s) cm^-1^; ESI-MS m/z: 1027.05, [M-ClO_4_]^+^; Anal. Calcd (%), for C_56_H_41_Cl_8_MnN_3_O_17_: C 56.63, H 3.469, N 3.65; Found. C 56.54, H 3.49, N 3.73. (Spectra of ESI-MS See in Supplementary Figure 25).

#### Synthesis of [Zn(LY)_2_(ClO_4_)_2_] (4)

The same method was employed by 2, yield of 60%. IR(KBr):(-NH) 3438(m), (Ar-H) 2944(m), (C=O) 1608(m), (C=C) 1578, 1482, 1467(s), (C-O)1412, 1275(vs), (C-N) 1045(s) cm^-1^; ESI-MS m/z: 745.05, [M-ClO_4_]^+^ (Supplementary Figure 26); Anal. Calcd (%), for C_36_H_26_Cl_2_ZnN_2_O_14_: C 50.65, H 2.96, N 3.14; Found. C 50.81, H 3.10, N 3.32.

### X–ray crystallography

The instrument and the method of single-crystal X-ray diffraction analysis was performed as our previously reports [13, 55, 66]. The CCDC numbers for complexes **1**–**4** are CCDC 1543054-1543057.

### Materials and other methods

All the materials, instrumentation, and the detailed procedures for other experimental methods are described in supporting information.

## CONCLUSIONS

In summary, we have developed a facile concise synthesis of lysicamine from simple raw materials under mild conditions. This successful synthesis could shed light on the preparation of other oxoaporphine alkaloids.

Four new metal complexes **1**–**4** of lysicamine (LY) were made and fully characterized. The complexes exhibit *in vitro* cytotoxicity to selected tumour cells. In particular, **2** and **3** showed high antitumor activity to HepG2 and NCI-H460 cells. Mechanistic studies showed that **2** and **3** blocked cell cycle at the S phase by increasing the expression of p53, p21, p27, and Cdc25A, as well as decreasing the expression of cyclin A2, cyclin B1, cyclin D1, cyclin E1, CDK2, CDK6 and PCNA. Besides, **2** and **3** also induced cell apoptosis by up-regulating Bax, Apaf-1, cytochrome *c* and down-regulating c-myc and Bcl-2. In addition, **2** and **3** also promoted ROS production, increased intracellular Ca^2+^, enhanced Δψ loss, and triggered caspase-3/-8/-9 activation. These results suggested that **2** and **3** mediated apoptosis via the caspase-dependent mitochondrial pathway. Gene chip experiments showed that **2** notably changed the expression levels of genes associated with death receptors, and indicated that **2** also induced apoptosis via the death receptor pathway. Furthermore, **2** inhibited HepG2 tumour growth *in vivo*, and showed better safety profile than cisplatin. In conclusion, this work confirmed that **2** and **3** had high antitumor activity towards HepG2 cells, and that **2** could be a promising candidate to be further developed into an effective antitumor agent.

## SUPPLEMENTARY MATERIALS FIGURES AND TABLES




