# Supplementary material for: Facile total synthesis of lysicamine and the anticancer activities of the RuII, RhIII, MnII and ZnII complexes of lysicamine
Source: Oncotarget. 2017 Jul 26;8(35):59359–75. doi: 10.18632/oncotarget.19584 (PMC5601738; doi:10.18632/oncotarget.19584)
Supplement: Supplementary file 1 [file oncotarget-08-59359-s001.pdf]

Facile total synthesis of lysicamine and the anticancer activities of the Ru^{II}, Rh^{III}, Mn^{II} and Zn^{II} complexes of lysicamine

SUPPLEMENTARY MATERIALS

Materials and instrumentation

All the chemical reagents used in the experiments were of analytical grade and commercially available. EB and AO were purchased from Solar Technologies, Inc. Propidium iodide (PI), MTT assay kit, were purchased from Sigma-Aldrich. Bax, Bcl-2, c-myc, CDK2, CDk6, Cdc25A, ATR, Chk1, p53 and PCNA were purchased from Abcam (U.S.A.). CyclinA2, CylinD, CyclinB, Apf-1 and cytochrome C, p21, and p27 were purchased from Cellsignaling (U.S.A.). CaspGLOW™ Fluorescein Active Caspase-3/-8/-9 Staining Kit from BioVision. All tumour cell lines were obtained from the Shanghai Institute for Biological Science (China). Purity of complexes **1**–**4** and LY were >98%, and dissolved in DMSO for the preparation of stock solution at a concentration of 2.0×10^{-3} M. TBE: Tris-boric acid-EDTA buffer solution was prepared using double distilled water.

The instrumentation used in this study were introduce in our previously reports [1-3].

Chemical stability test

The stability of **1**–**4** in PBS solution was carried out by UV-vis spectroscopy. The stock solution of **1**–**4** (2×10^{-3} M) was diluted to 2×10^{-5} M used TBS solution, the UV-vis absorbance of **1** and **2** at 0 h, 2 h, 4 h, 8 h, 12 h and 24 h were measured in 200~600 nm, respectively. Before the next testing, the measured sample of **1**-**4** were stored at 25°C in the dark for the indicated times. The chemical stability of **2** and **3** was further confirmed by HPLC. **2** and **3** in aqueous solution with 1.0 mg·mL^{-1} was analyzed by HPLC at 0 h and 24 h respectively, with reversed-phase C18 column and methanol/H_2O (80:20) mobile phase.

MTT assay

The cells BEL-7404, HepG2, NCI-H460, T-24 and HL-7702 were purchased from Shanghai Cell Bank in Chinese Academy of Sciences. Cells were cultured in DMEM or RPMI-1640 medium at 37 °C in a humidified atmosphere with 5% CO_2/95% air. The stock solution of **1**–**4** and **LY** prepared as 2.0×10^{-3} mol/L DMSO, and diluted to 20 M by PBS buffer when used. Cisplatin was dissolved in 0.9% sodium chloride solution and used as positive controls.

Cells were seeded in 96-well plates with 5.5 $\times10^{3}$/180 µL per well for 24 h to reach 70% confluence, 20 µL of various concentrations of tested compounds were added to each well, each concentration was provided with 5 parallel holes. All the cells were incubated with test compounds for 48 h before 10 µL of MTT (5 mg/mL in PBS) was added to each well, and cells were incubated for another 4 h. after removed the medium, 150 µL DMSO were added to dissolve the formazan crystals, and the absorbance was recorded by enzyme labelling instrument with 490 nm/630 nm double wavelength measurement. The antitumor inhibition rate was evaluated based on the percentage of cells survival compared with the untreated cells. The IC_{50} value was defined as complex concentration killing 50% cells in comparison with control cells, which calculated by the Bliss method (n = 5). All tests were repeated in at least three independent trials.

Uptake of rhodium in HepG2 cells

About 1×10^{6} HepG2 cells were treated with **2** (7.0µM) and **3**(14.0 µM) for 24 h, respectively. Then the treated cells were harvested and dissolved in 1 M NaOH (1 mL) and diluted with 2% (v/v) HNO_3(5 mL) for determining the whole cell Rh(III), Mn(II) and Pt(II) content. The cells' nucleus fraction and mitochondria fraction were isolated using the FractionPREP kit from BioVision according to the instructions and digested with HNO_3, and then diluted with double-distilled water to obtained a final solution with concentration of 5% HNO_3 (5 mL). The amount of each metal were determined by plasma-mass spectrometry (ICP-MS).

Cell cycle analysis

Cell cycle progression was determined by flow cytometric analysis. About 5×10^{6} cells/well HepG2 and NCI-H460 cells were treated with **2** and **3** at various doses (3.5, 7.0, 14.0µM for **2**; 7.0, 14.0 and 28.0 µM for **3**) for 24 h. After incubated with **2** and **3**, cells were trypsinized, collected, and fixed in ice-cold 75% ethanol overnight at -20 °C. The next day, the fixed cells were washed with ice-cold PBS and resuspended in 0.5 mL of PBS containing 100 µg/mL RNase, 50 µg/mL PI in the dark for 5–10 min. The cell cycle distribution was analyzed by FACS Calibur flow cytometer (BD) and calculated using ModFIT LT software (BD).

Apoptosis assay

The method of incubation HepG2 and NCI-H460 cells with **2** and **3** were the same as the cell cycle analysis assay.

After treated, the cells were collected, and suspended in the annexin-binding buffer (5 × 10^5 cells/mL), then treated with annexin V-FITC and PI for 1 h at room temperature in the dark and immediately analyzed by flow cytometry.

Hoechst33258 assay

The morphology characters of apoptosis of HepG2 cells treated with **2** and **3** were carried out by Hoechst 33258 staining. About 1×10^6 cells were seeded in six-well plates, and treated with **2** (3.5, 7.0 and 14.0 μM) and **3** (7.0, 14.0 and 28.0 μM) for 24 h. The cells were fixed with carnoy-fixe solution for 10 min at room temperature and then stained with Hoechst 33258 for another 10 min in the dark. After washing three times with PBS, the HepG2 cells' morphological features of apoptosis were captured by fluorescence microscope with excitation wavelength of 330-380 nm. Apoptotic cells were defined based on the nuclear morphology changes such as chromatin condensation and fragmentation.

Measurement of oxygen species (ROS), intracellular Ca^{2+} and mitochondrial membrane potential (ΔΨm)

HepG2 cells at about 1×10^6 cells/well were plated onto 6-well plates and incubated at 37 °C for 24 h, then cells were exposed to **2** (3.5, 7.0 and 14.0 μM) and **3** (7.0, 14.0 and 28.0 μM) for 24 h. These cells were harvested and then resuspended in 1mL of DCFH-DA (100 μM) for ROS determination [4], in 1 mL Fluo-3 AM (0.5μM) for intracellular Ca^{2+} concentration [4, 5], in 1 mL JC-1 (5μg/mL) [6] or rhodamine (0.5 μg/L)[5] for ΔΨm measurements. Then the cells incubated for another 30 min, moved the culture solution, washed with PBS three times and re-suspended in 2 mL PBS, analyzed by flow cytometry or fluorescence microscope. The emission fluorescence for ROS was 525 nm, excitation wavelength was 488 nm; for Ca^{2+}, emission wavelengths was 526 nm, excitation wavelength was 506 nm; for monomer JC-1, emission wavelengths was 530 nm, excitation wavelength at 490 nm, for J-aggregates, emission wavelengths was 590 nm, excitation wavelength at 525 nm.

Caspase- 3, -8 and -9 activity determinations by flow cytometry

CaspGLOW fluorescein active caspase-3/-8/-9 staining kit was used in this assay. HepG2 and NCI-H460 cells treated with IC_{50} value of **2** and **3** for 24 h, and harvested at a density of 1 × 10^6 cells/mL, and then resuspended in 300 μL volume with PBS contains 1 μL of caspase-3 inhibitor (FITC-DEVD-FMK), caspase-8 inhibitor (FITC-IETD-FMK) or caspase-9 inhibitor (FITC-LEHD-FMK), respectively, and incubated for another 1.0 h at 37 °C in 5% CO_2 incubator. The cells were harvested by centrifugation, then examined used a FACSAria II flow cytometer equipped. The results were represented as the percent change on the activity comparing with the control.

Cell cycle-, apoptotic-associated proteins used western blotting analysis

Extraction of total protein

After HepG2 cells incubated with **2** (3.5, 7.0, 14.0 μM) and **3** (7.0, 14.0, 28.0 μM) for 24 h, cells were harvested and lysed used 149μL RIPA and 1μL PMSF on ice. The suspension sample was centrifuged at 12 000 rpm at 4 °C for 10 min, the supernatant liquid (total protein) was extracted, the total protein stocked in–80 °C refrigerator before used. The total protein absorbance value of the 562 nm was measured by the enzyme marker, and the protein concentration was calculated according to the standard curve [7].

Determination of cell cycle and apoptotic related proteins

The quantitative determination of protein according to the manufacturer's instructions. 25μL SDS-PAGE protein sample buffer was added to 100 μL total protein liquid and then was boiled for 5 min. 10 μL protein sample was loaded onto 10% SDS-PAGE gels and then transferred onto a PVDF membrane. The membrane was blocked with 5% BSA in TBST buffer for more than 2 h. After moving the TBST buffer, membranes incubated with an primary antibodies in TBST overnight at 4 °C and then washing with TBST three times, the membranes incubate with anti-mouse or anti-rabbit secondary antibodies (anti-CDK2, CDK6, Cyclin A2, Cyclin D1, Cyclin B1, PCNA, p53, pp21, p27, Apaf-1, cytochrome*c*, caspase-3/-8/-9, c-myc, Bax, Bcl-2, PARP, and β-actin) for 1 h. The protein bands were visualized using chemiluminescence substrate.

Gene expression by a panel of genes for RT-qPCR array

The DNA chip analysis was carried out according to our previously report [3]. Differential expression profiles of cell cycle and apoptosis-related genes were analyzed using the human cell cycle PCR array(PAHS-020Z) and human apoptosis PCR array(PAHS-012Z), which implemented by Kangchen Biotech (Shanghai, China).

HepG2 cells incubated with **2** (7.0 μM) for 24 h, then 1 mL trizol was added to 15 cm^2 adherent cells, RNA was extracted according to standard protocols and converted to first strand cDNA using the RT2 First Strand Kit. Then added with RT2 SYBR Green qPCR Master Mix and each of the respective forward and reverse primers and

RT-qPCR was performed. The threshold cycle (Ct) values for all the genes on each PCR Array were calculated using the instrument specific software, and the fold-changes in gene expression for pairwise comparison were calculated using the $2^{-\Delta Ct}$ method.

DNA binding experiment

In the DNA binding experiment, ct-DNA was stock at 4 °C with 2×10^{-3} M (solute in TBS buffer) for no more than 3 days before used. Complexes and LYwere all prepared as 2×10^{-3}M DMSO stock solutions, the DMSO is limited in 1% in final working solutions.

CD absorption spectrometry assay

In the CD absorption spectrometry, 150 μL2×10^{-3} M ct-DNA and 2850 μL TBS were added to a cuvette, the ct-DNA CD spectrum as control. LY, **2** and **3** were added into the ct-DNA solution gradually, with the [compounds] / [DNA] ratio were 0:10, 0.5:10, 1:10, 1.5:10, 2.0:10 and 2.5:10. The working solution was incubated for 10 min after each addition and then its CD spectrum was recorded at 100 nm/min scan rate. The CD signals of the TBS were subtracted as the background.

Agarose gel electrophoresis assay

In plasmid DNA unwinding experiments, 1 μL 0.5 μg/μL supercoiled pBR322 DNA was treated with different concentration of LY, **2** and **3** in TBE buffer at 37 °C in the dark for 4 h. Then each sample mixed with loading buffer in 5:1(v/v), 12μL sample analyzed by 1% agarose gel electrophoresis at 5 V/cm in 1×TBE buffer solution. Finally, the gel was then stained by EB (0.5 μg/mL) for 20 min and photographed via a BIO-RAD imaging system under a UV-Vis transilluminator.

In vivo tumour growth inhibition experiment

Animal used

Animals were supplied by Guangxi Medical University Laboratory Animal Centre (Guangxi, China, approval no. SCXK 2014-0002 and SYXK 2014-0003), and the animal experiment were carried out at there. KM mice, half male and female, 20-23g, 5-6 weeks old for acute toxicity test. BALB/c nude mice, male, 19-21 g, 5-6 weeks old for antitumor xenograft experiment. Animals were housed at a sterile environment with conditions of constant photoperiod (12 h light/12 h dark at 25–28 °C and 45%–65% humidity), in addition, BALB/c nude mice housed in individual ventilated caging system (IVC Rack).

Maximumt tolerated dose (MTD)

The MTD was determined using male and female KM mice. The large concentration of **2** in 10% (v/v) DMSO solution (0.57mg/mL) was single intraperitoneal injected to 10 KM mince with 0.4 mL/10 g (*i.e.*, 22.8 mg/kg). The body weight of mice was evaluated daily for the first 5 days and then twice a week thereafter. The MTD was defined as the largest administered dose of drug and route cause a mean body weight loss≤20%, no animal death and the reversible and temporary toxicities [8].

HepG2 xenograft models

The xenograft tumour model was established in the same way in our previous reports [3, 9]. The HepG2 bearing mice were randomized into four groups (n=6): control group, **2**-treated group (7.6 mg/kg and 3.8 mg/kg) and cisplatin-treated group (2.0 mg/kg, dissolve in saline). The HepG2 xenograft mice were induced by intraperitoneal injection of drugs every two days, and the tumour volumes and body weight was recorded every three days. The percent weight loss or gain was calculated using the initial weight as a reference. Tumour growth trend was describe by tumour size with volume=$(w^2 l)/2$, where w is the width and l is the length in mm of tumour [10]. Percent tumour growth inhibition after initiation of treatment with **2** and cisplatin were calculated by TGI= 100 × 1−(tumour volume $_{final}$ −tumour volume $_{initial}$ for drug-treated group)/(tumour volume $_{final}$ −tumour volume $_{initial}$ for control group) [8].

REFERENCES

1. Wei JH, Chen ZF, Qin JL, Liu YC, Li ZQ, Khan TM, Wang M, Jiang YH, Shen WY, Liang H. Water-soluble oxoglaucine-Y(III), Dy(III) complexes: *in vitro* and *in vivo* anticancer activities by triggering DNA damage, leading to S phase arrest and apoptosis. Dalton Trans. 2015; 44: 11408–11419.
2. Qin QP, Liu YC, Wang HL, Qin JL, Cheng FJ, Tang SF, Liang H. Synthesis and antitumor mechanisms of a copper(II) complex of anthracene-9-imidazolinehydrazone (9-AIH). Metallomics. 2015; 7: 1124–1136.
3. Chen ZF, Qin QP, Qin JL, Zhou J, Li YL, Li N, Liu YC, Liang H. Water-soluble ruthenium(II) complexes with chiral 4-(2,3-dihydroxypropyl)-formamideoxoaporphine(FOA): *in vitro* and *in vivo* anticancer activity by stabilization of G-Quadruplex DNA, inhibition of telomerase activity, and induction of tumor cell apoptosis. J Med Chem. 2015; 58: 4771–4789.
4. Khan M, Ding C, Rasul A, Yi F, Li T, Gao H, Gao R, Zhong LL, Zhang K, Fang XD, Ma TH. Isoalantolactone induces reactive oxygen species mediated apoptosis in pancreatic carcinoma PANC-1 cells. Int J Biol Sci. 2012; 8: 533–547.
5. Zou HH, Wang L, Long ZX, Qin QP, Song ZK, Xie T, Zhang SH, Liu YC, Lin B, Chen ZF. Preparation of 4-([2,2’:6’,2’’-terpyridin]-4’-yl)-N, N-diethylaniline Ni^{II} and Pt^{II} complexes and exploration of their *in vitro* cytotoxic activities. Eur J Med Chem. 2016; 108: 1–12.
6. Binet MT, Doyle CJ, Williamson JE, Schlegel P. Use of JC-1 to assess mitochondrial membrane potential in sea urchin sperm. J Exp Mar Biol Ecol. 2014; 452: 91–100.
7. Liao WZ, Lu YJ, Fu JN, Ning ZX, Yang J, Ren JY. Preparation and characterization of Dictyophora indusiata polysaccharide-Zinc complex and its augmented antiproliferative activity on human cancer cells. J Agric Food Chem. 2015; 63: 6525−6534.
8. Sampson PB, Liu Y, Forrest B, Cumming G, Li SW, Edwards NK, Laufer R. The discovery of Polo-like kinase 4 inhibitors: identification of (1 R, 2 S)-2-(3-((E)- 4-(((cis)-2, 6-dimethylmorpholino) methyl) styryl)-1 H-indazol-6-yl)-5’-methoxyspiro [cyclopropane-1, 3’-indolin]-2’-one (CFI-400945) as a potent, orally active antitumor agent. J Med Chem. 2014; 58: 147–169.
9. Nakano D, Ishitsuka K, Kamikawa M, Matsuda M, Tsuchihashi R, Okawa M, Okabe H, Tamura K, Kinjo J. Screening of promising chemotherapeutic candidates from plants against human adult T-cell leukemia/lymphoma(III). J Nat Med. 2013; 67: 894–903.
10. Cheng JJ, Khin KT, Davis ME. Antitumor activity of β-cyclodextrin polymer-camptothecin conjugates. Mol Pharm. 2004; 1: 183–193.

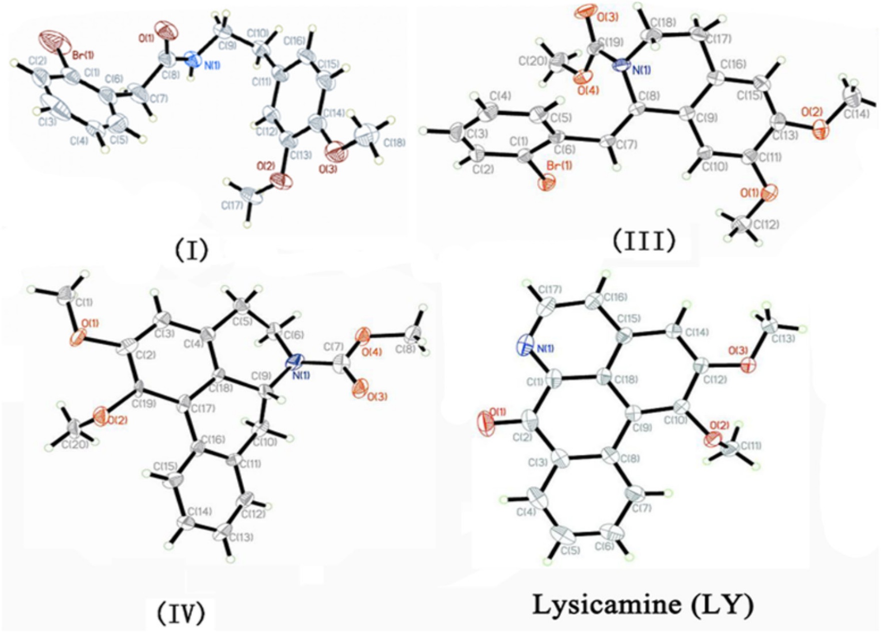

Supplementary Figure 1: The crystal structures of compound (I), (III), (IV) and Lysicamine (LY).

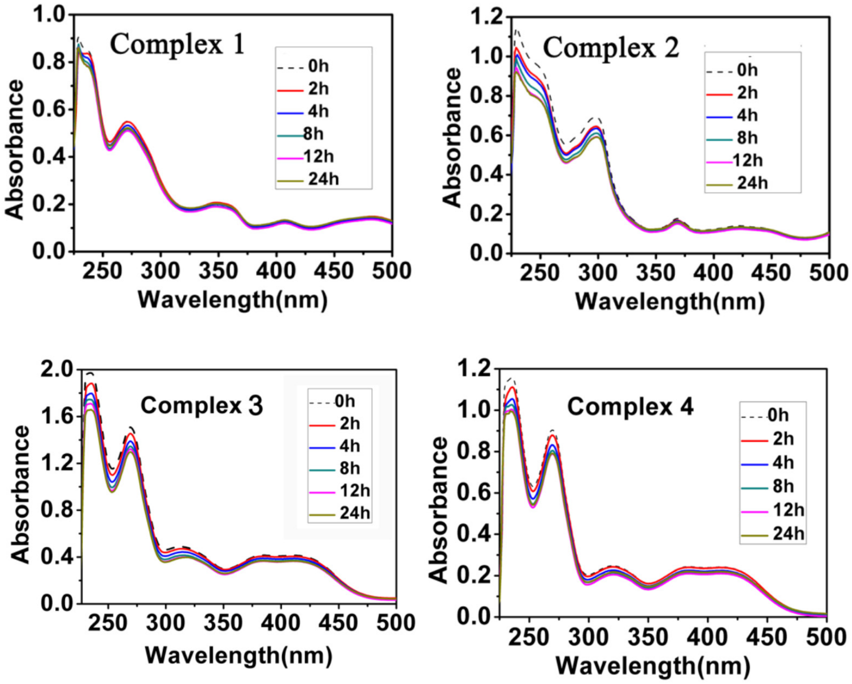

Supplementary Figure 2: Solution stability of complexes 1-4 in tris buffer solution examined by UV-vis spectra.

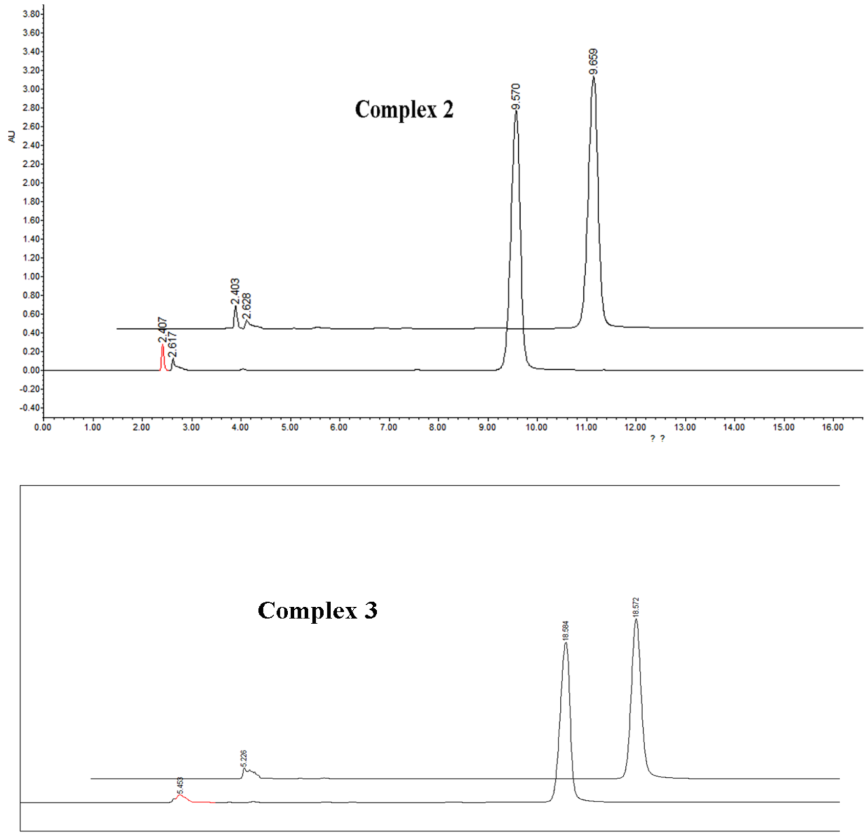

Supplementary Figure 3: HPLC spectra of 2 and 3 in aqueous solution (1 mg/mL) with time 0 h and 24 h respectively. Column: reversed-phase C18 column (YMC HPLC COLUMN, 250×4.6mm I. D.). Column temperature: 35. Mobile phase: Methanol/H_2O (80:20). Flow rate: 1.0 ml/min. Injection volume: 10 μL.

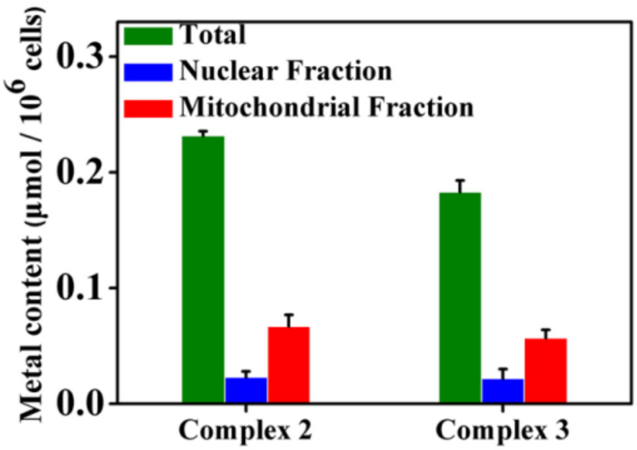

Supplementary Figure 4: Rhodium and manganese content in whole cell, nuclear and mitochondrial fractions were measured by ICP-MS. HepG2 were treated with **2** at 7.0 µM, **3** at 14.0 µM for 8 h at 37 °C. Data shown are mean values ±SD of three independent tests for each experiment.

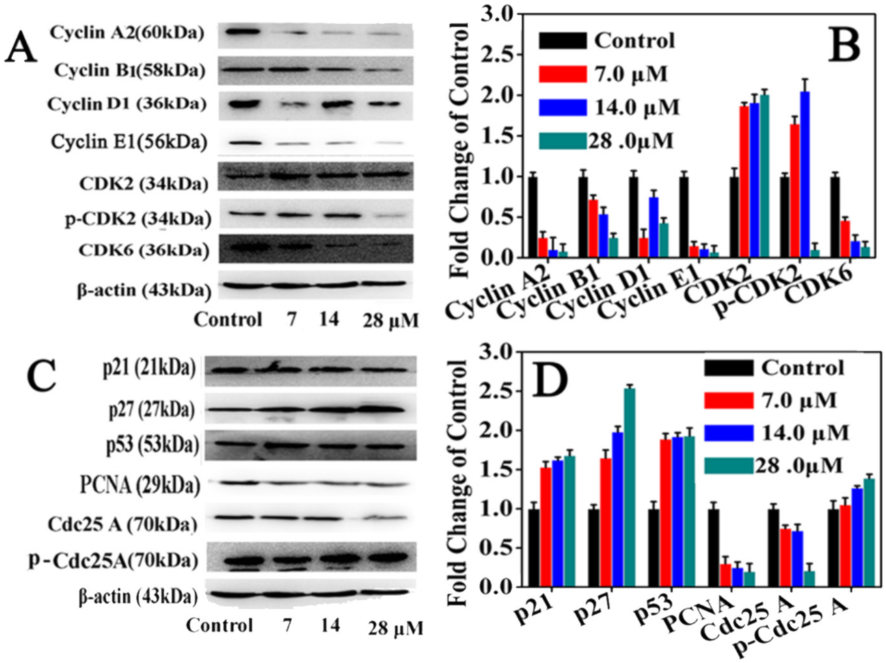

Supplementary Figure 5: (A, C) Effects of complex 3 treatment in HepG2 cells on cell cycle regulatory proteins at 7.0, 14.0 and 28.0 µM for 24 h, respectively. **(B, D)** The relative protein expression of each band = (density of each band/density of β-Actin band). Mean ± SD was from three independent measurements.

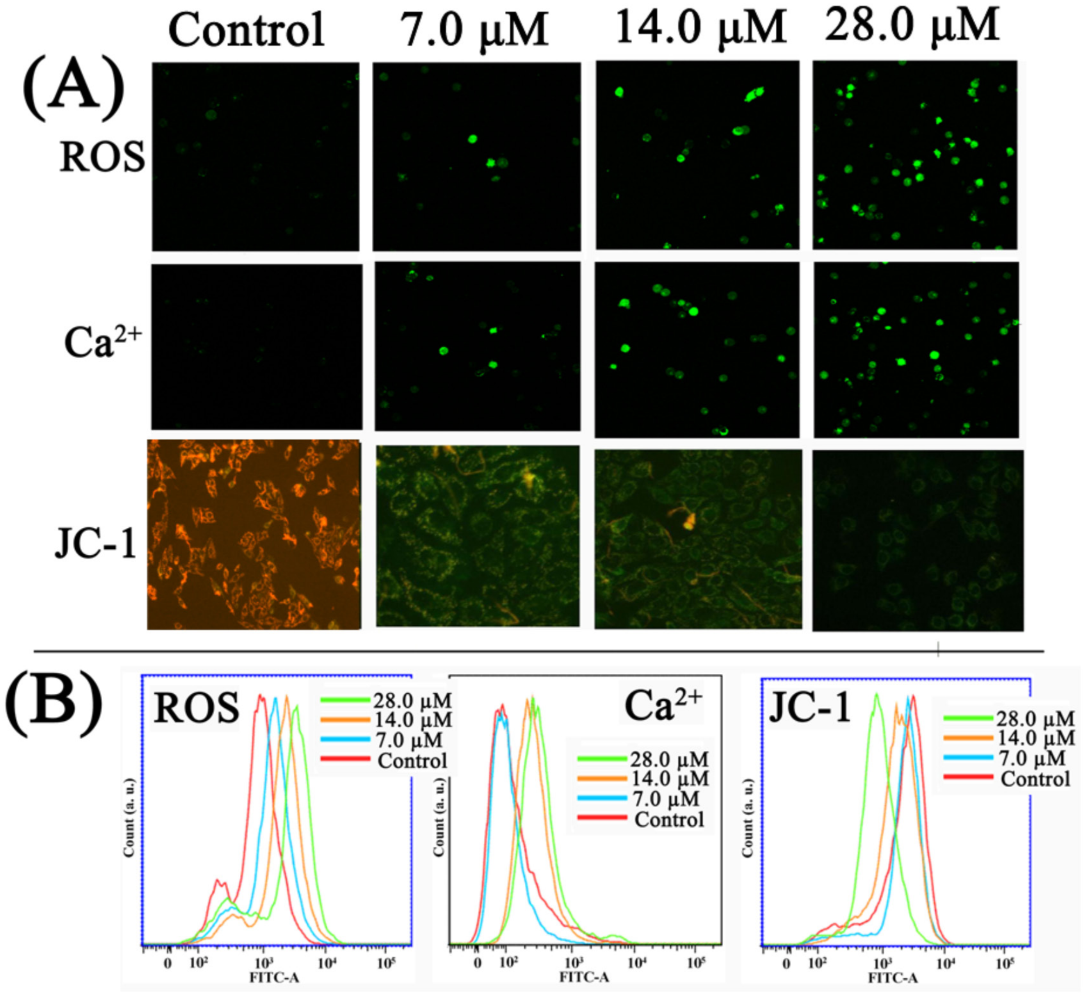

Supplementary Figure 6: The effect of 3 on the levels of ROS, intracellular Ca^{2+} and the loss of ΔΨm after Hep-G2 cells treated with 3 at 7.0, 14.0 and 28.0 μM for 24h, respectively. (A) The images of fluorescence microscope (magnification 100×) **(B)** The change of ROS, Ca^{2+} and ΔΨm examined by flow cytometry assay.

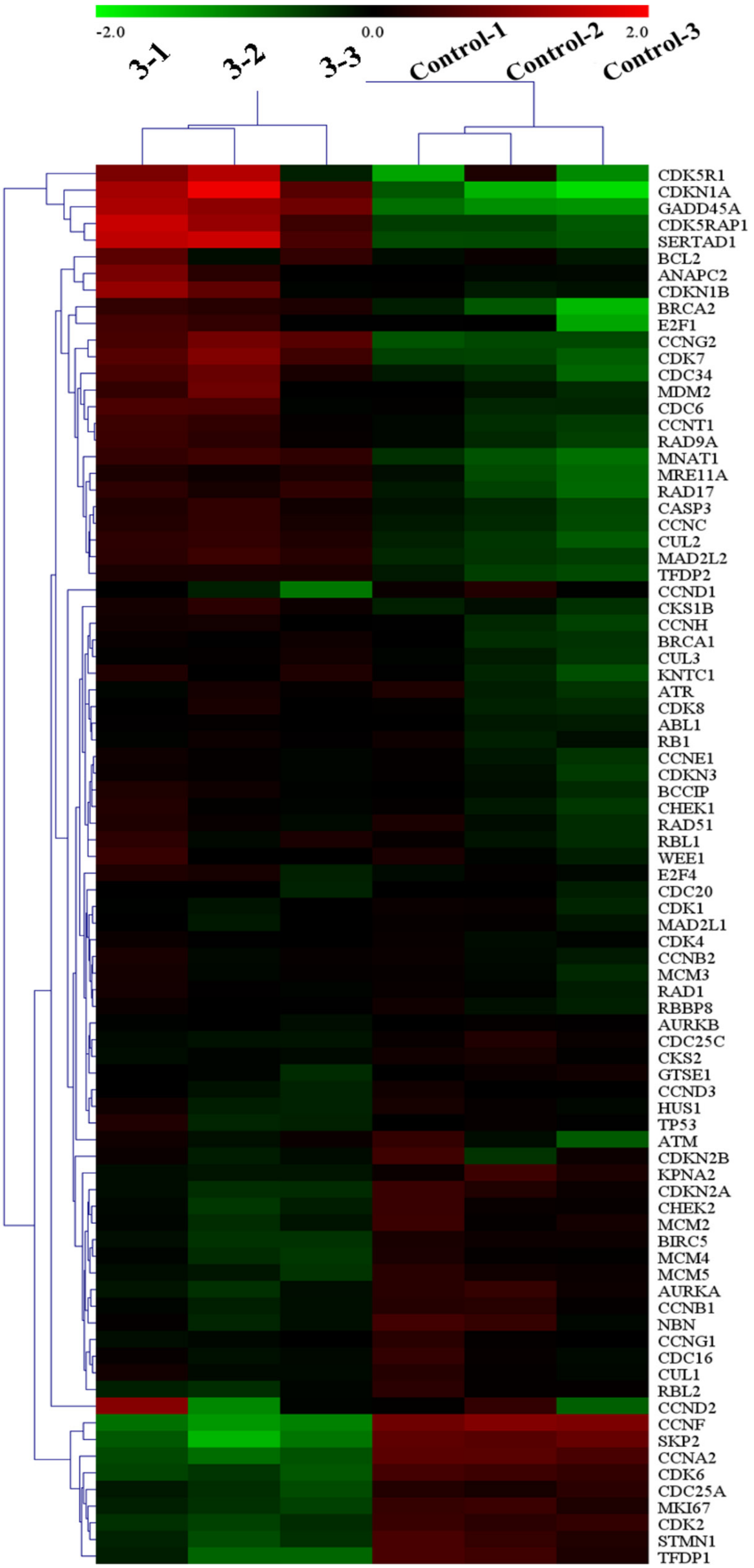

Supplementary Figure 7: The mRNA expression levels of cell cycle regulators genes in HepG2 cell after treated with 3 (14μM) for 24 h.

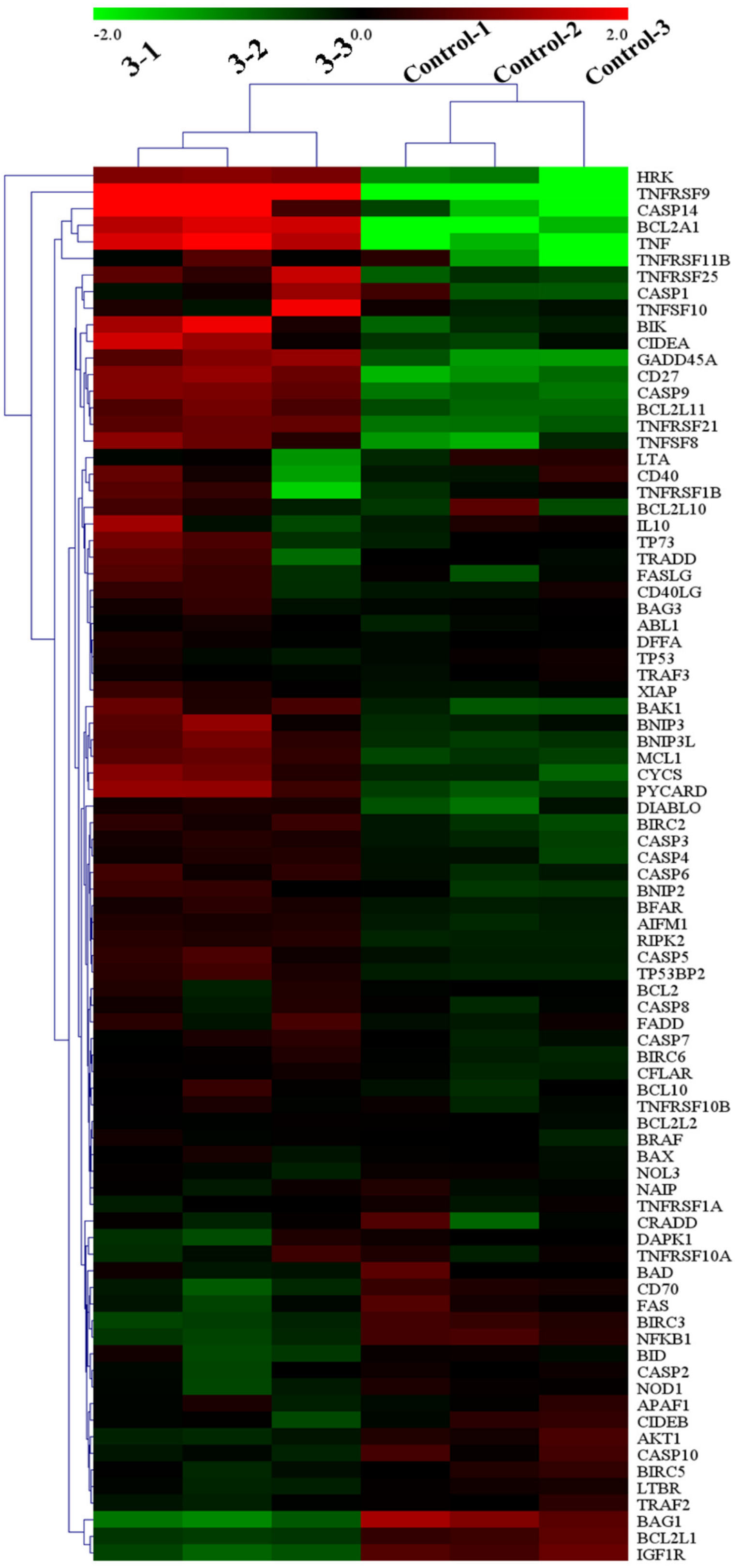

Supplementary Figure 8: The mRNA expression levels of apoptosis-related genes in HepG2 cells after treated with 3 (14μM) for 24 h.

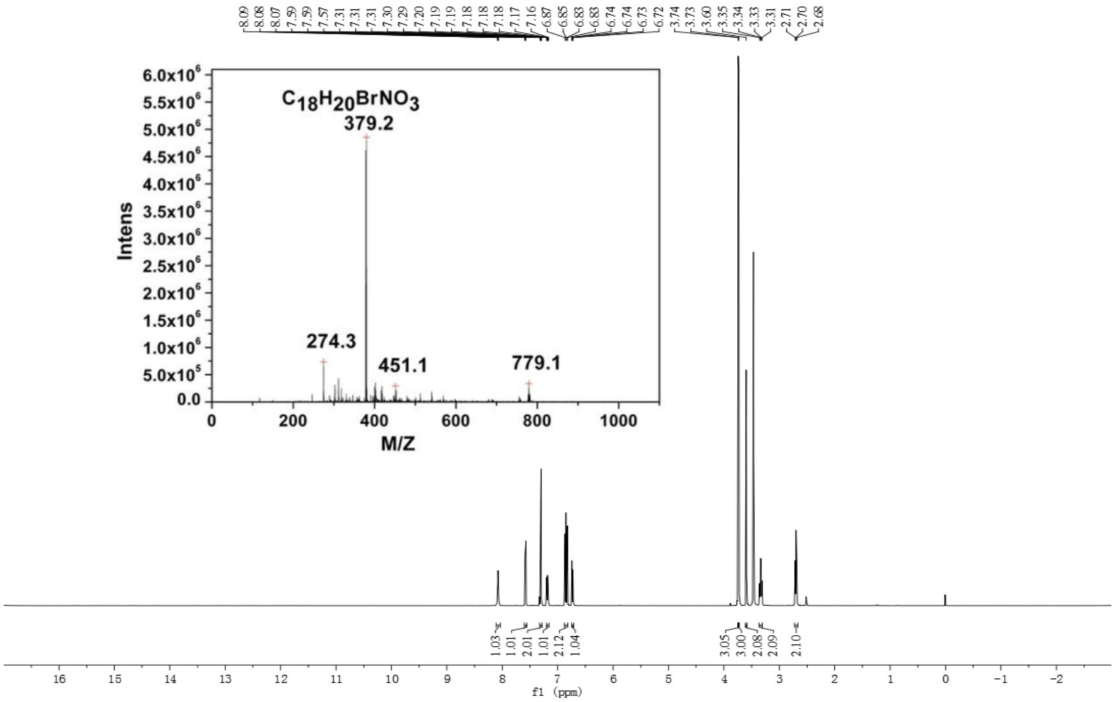

Supplementary Figure 9: ^{1}H NMR (600MHz, DMSO-d_6) and ESI-MS(inset) of compound (I).

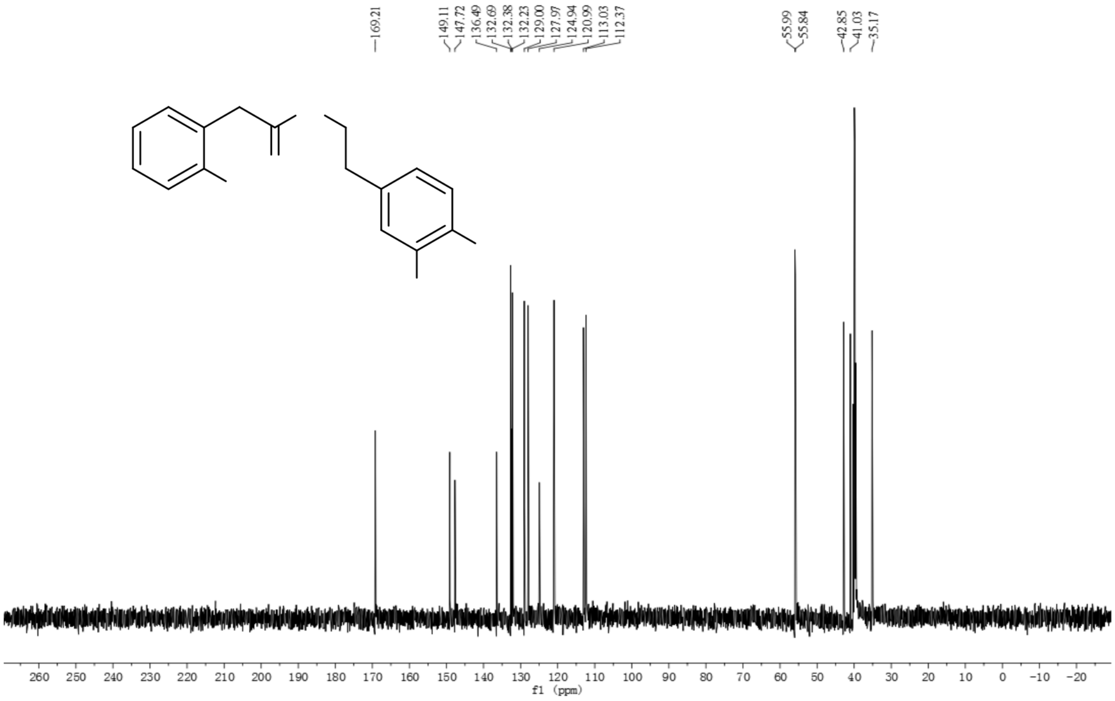

Supplementary Figure 10: ^{13}C NMR (600MHz, DMSO-d_6) of compound (I).

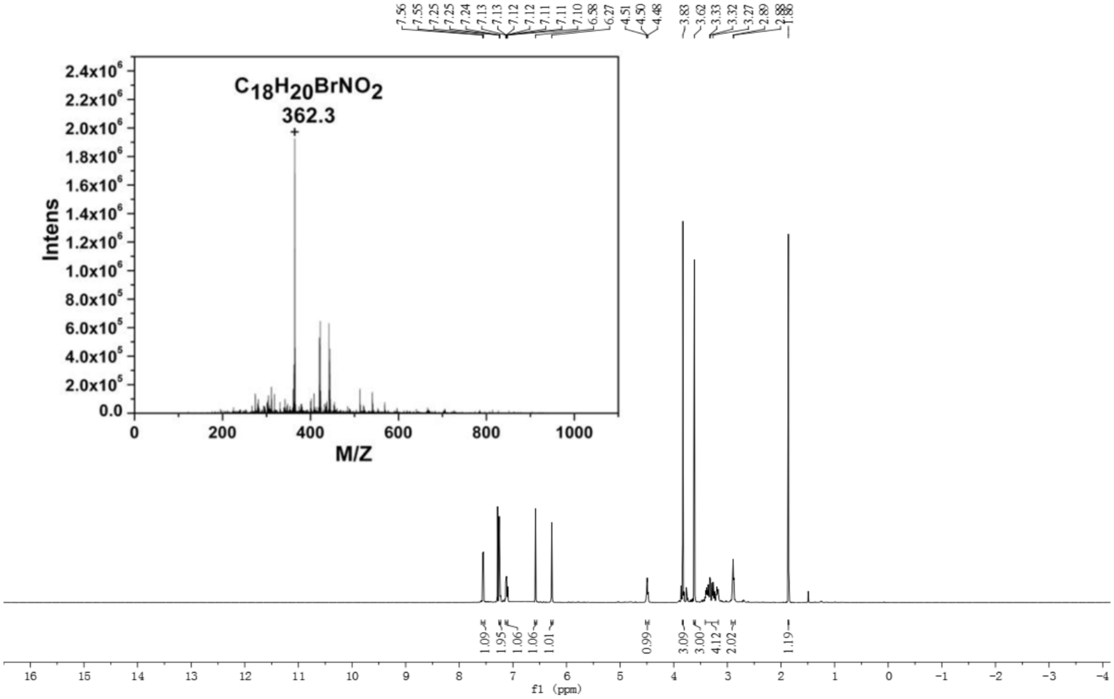

Supplementary Figure 11: ^{1}H NMR (600MHz, DMSO-d_6) and ESI-MS(inset) of compound (II).

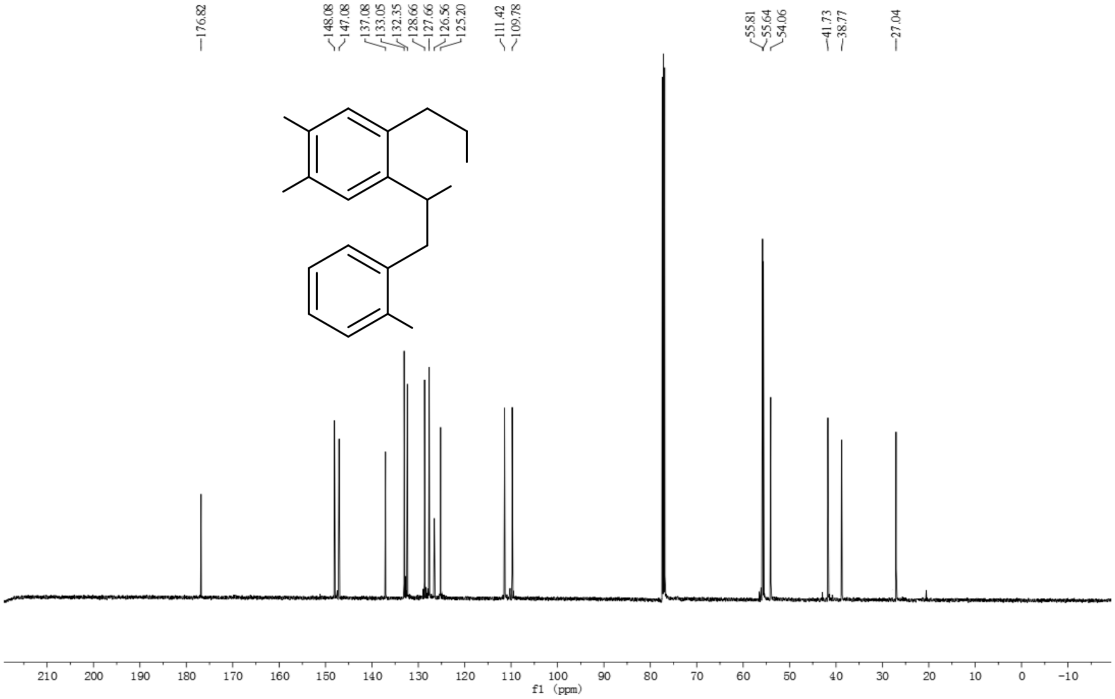

Supplementary Figure 12: ^{13}C NMR (600MHz, DMSO-d_6) of compound (II).

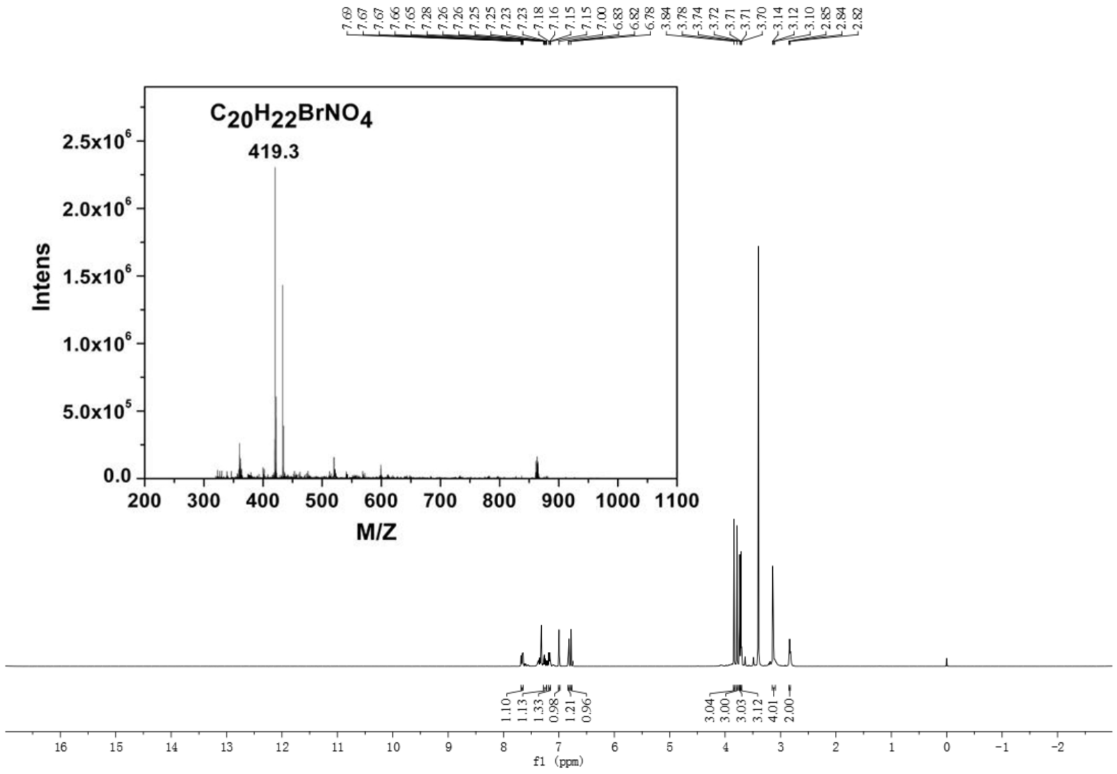

Supplementary Figure 13: ^{1}H NMR (600MHz, DMSO-d_6) and ESI-MS(inset) of compound (III).

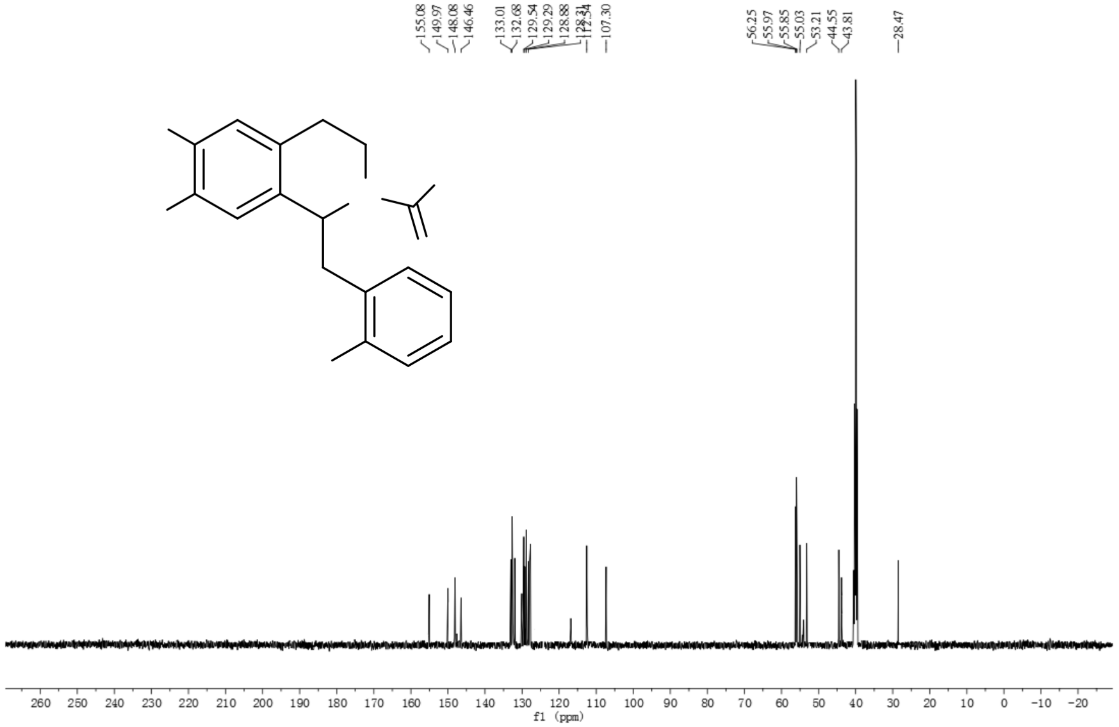

Supplementary Figure 14: ^{13}C NMR (600MHz, DMSO-d_6) of compound (III).

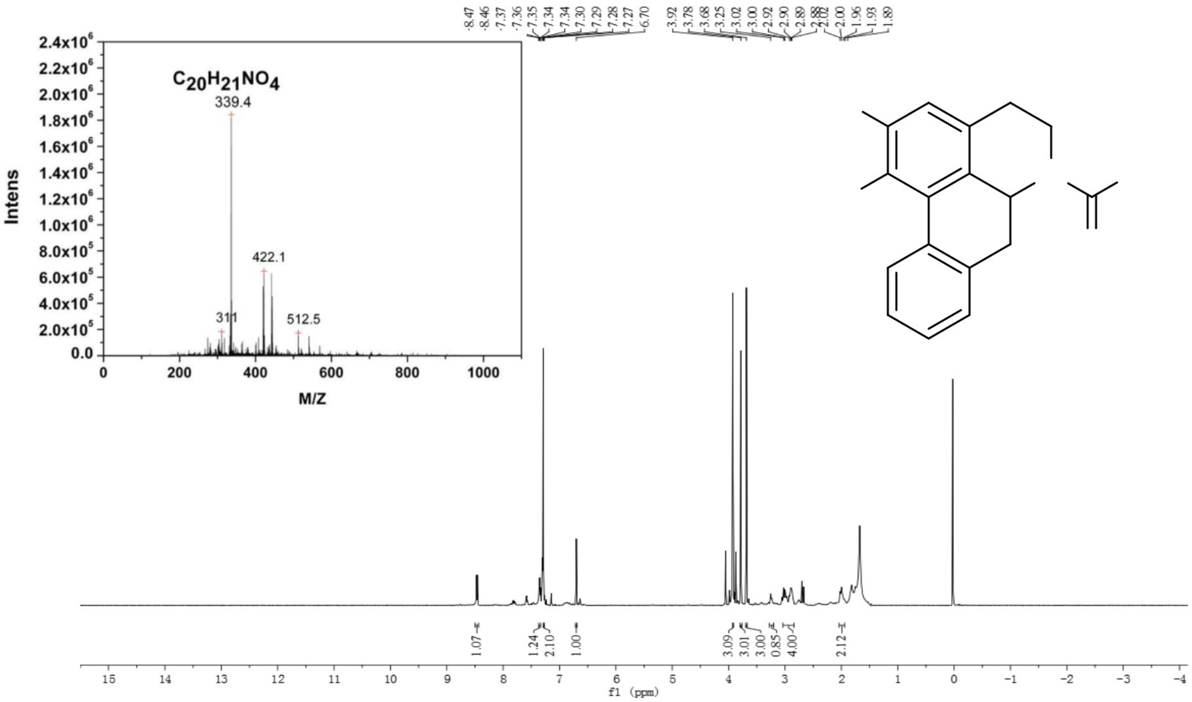

Supplementary Figure 15: ^{1}H NMR (600MHz, DMSO-d_6) and ESI-MS(inset) of compound (IV).

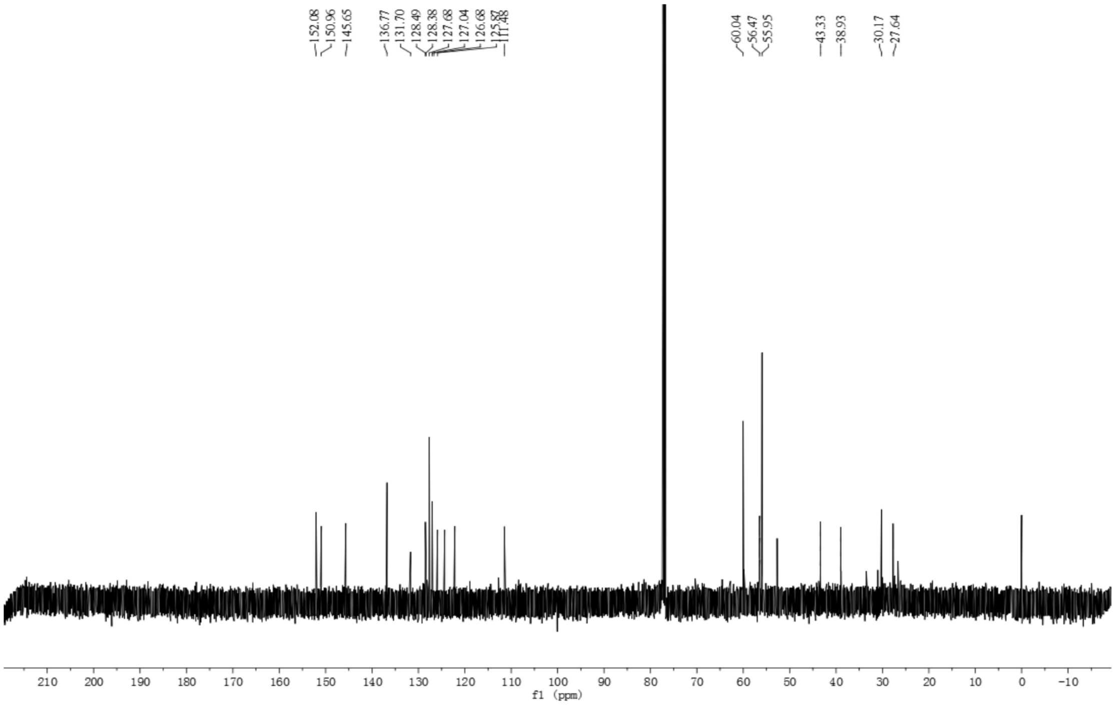

Supplementary Figure 16: ^{13}C NMR (600MHz, DMSO-d_6) of compound (IV).

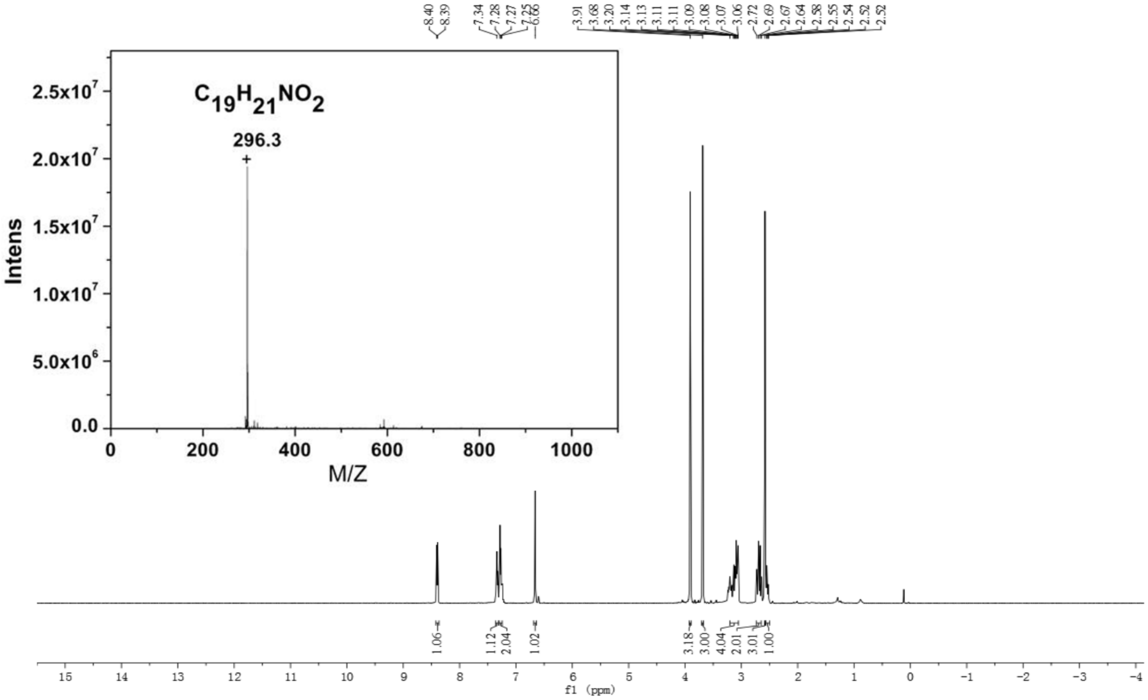

Supplementary Figure 17: ^{1}H NMR (600MHz, DMSO-d_6) and ESI-MS(inset) of compound (V).

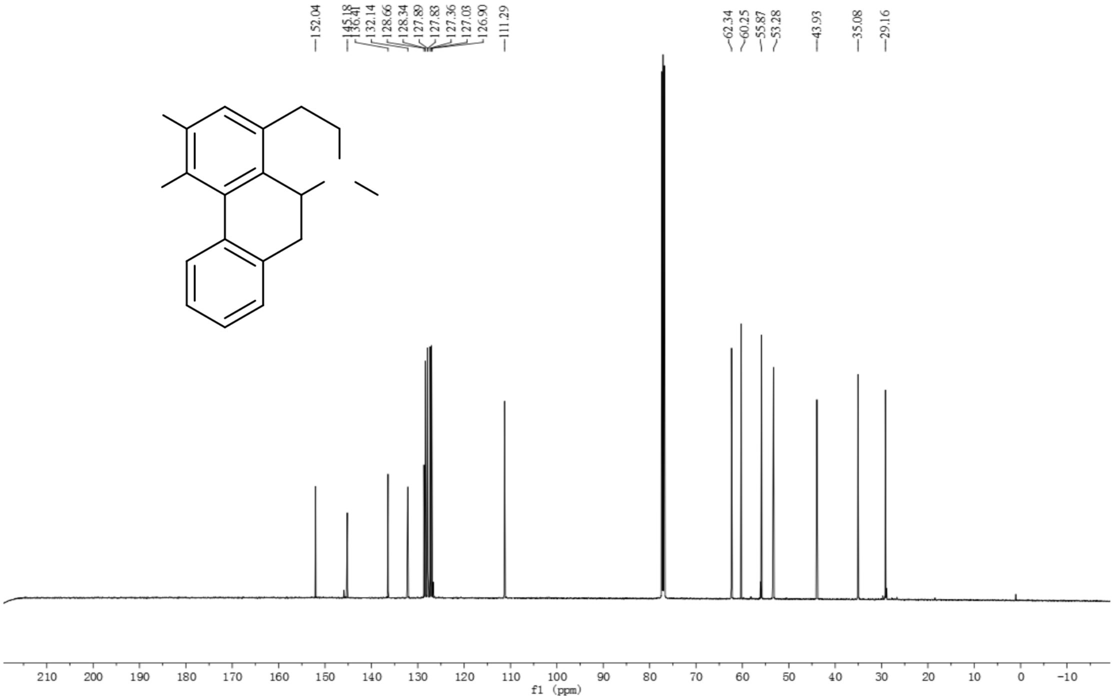

Supplementary Figure 18: ^{13}C NMR (600MHz, DMSO-d_6) of compound (V).

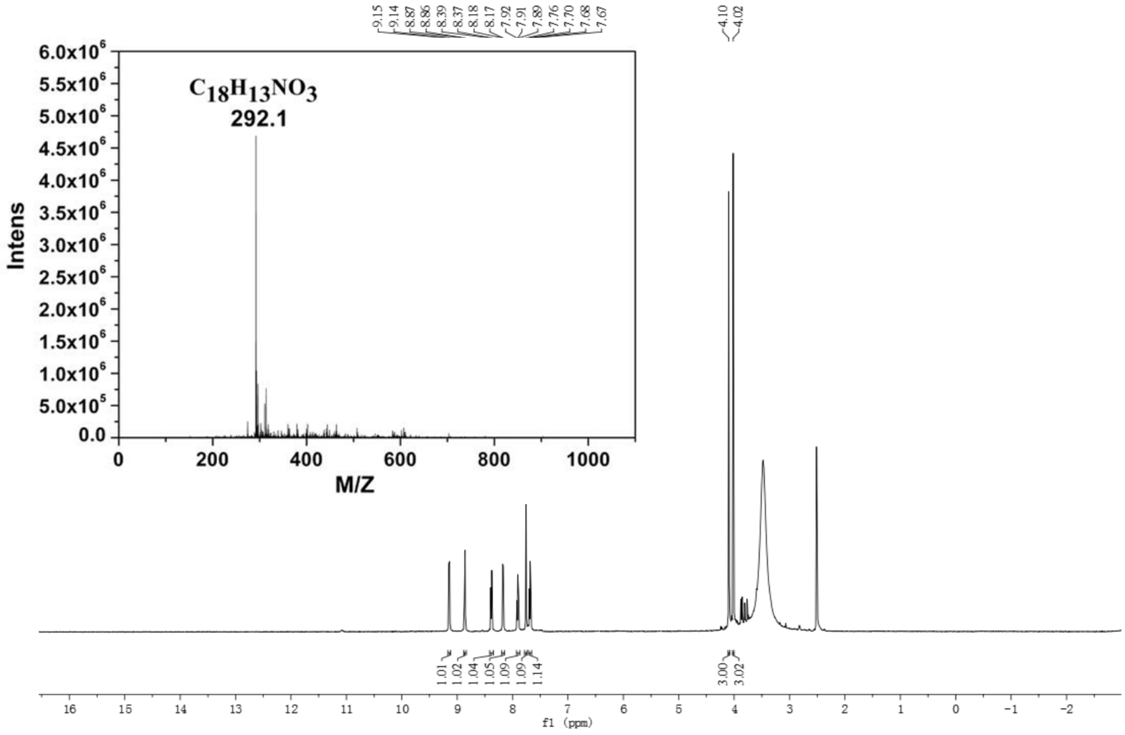

Supplementary Figure 19: **^{1}H NMR (600MHz, DMSO-d_6) and ESI-MS(inset)of lysicamine (IY).**

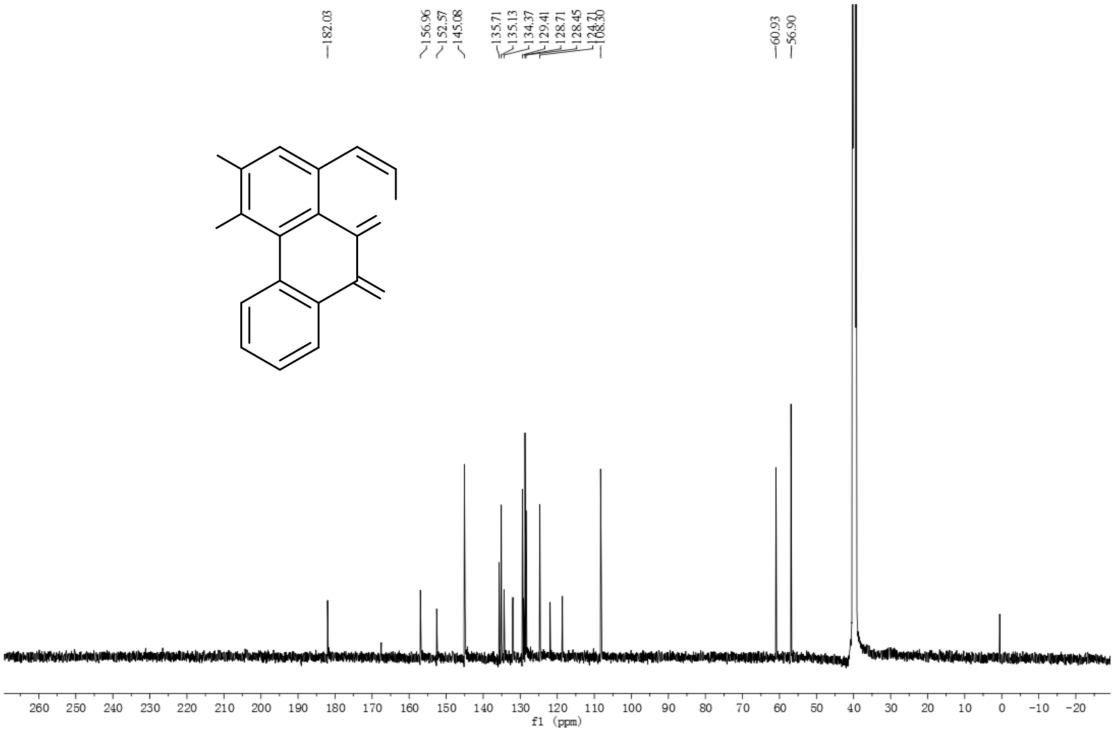

Supplementary Figure 20: **^{13}C NMR (600MHz, DMSO-d_6) of lysicamine (IY).**

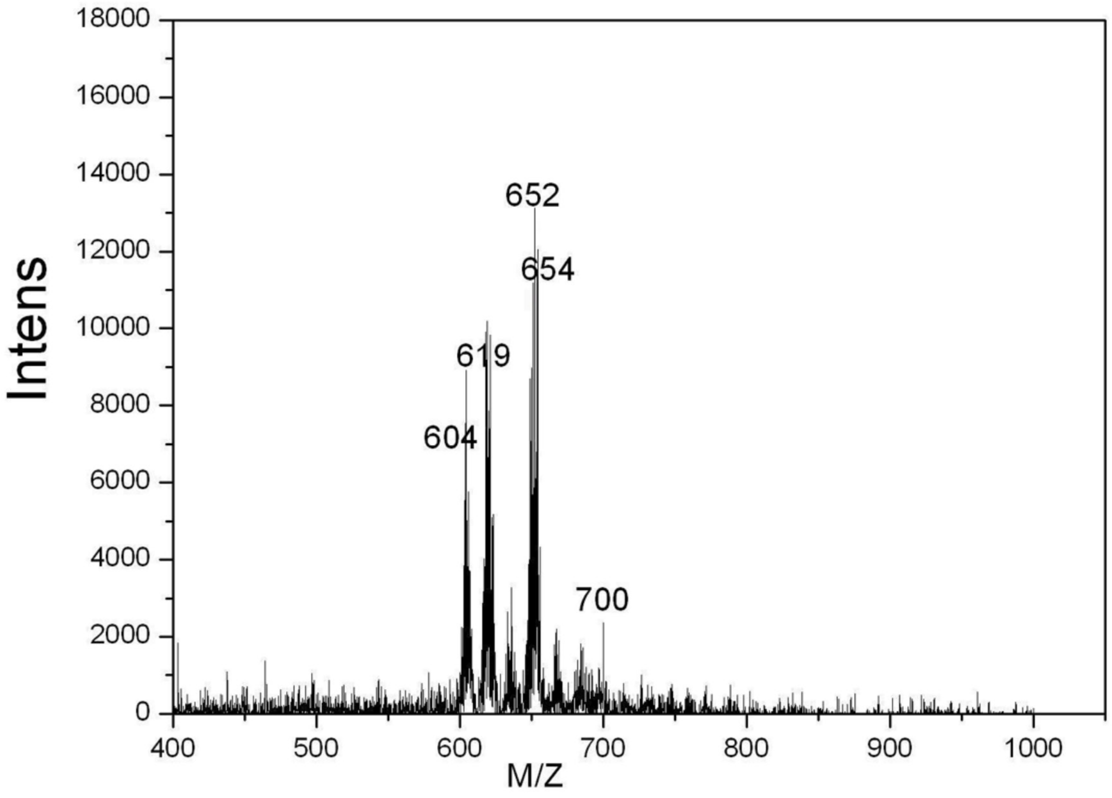

Supplementary Figure 21: ESI-MS analysis of complex 1.

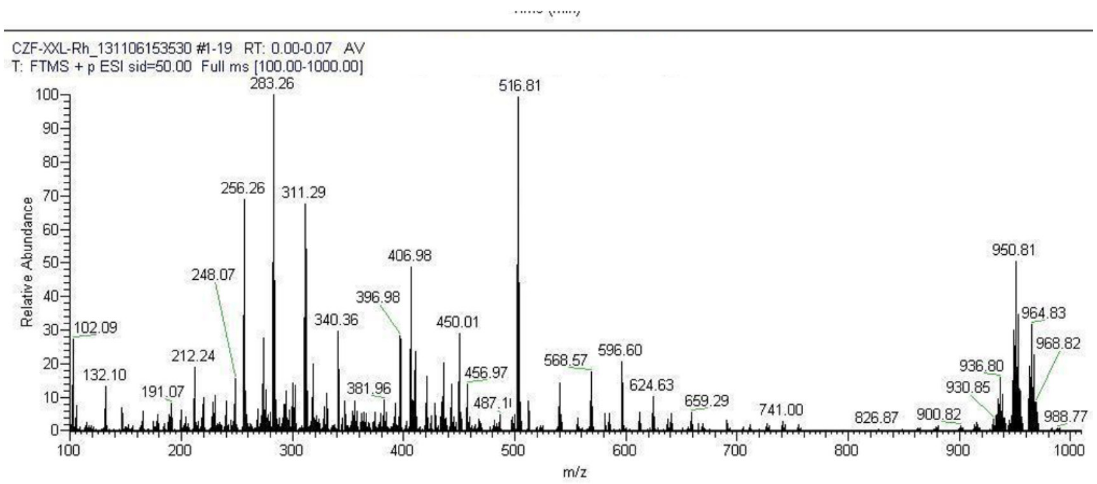

Supplementary Figure 22: ESI-MS analysis of complex 2.

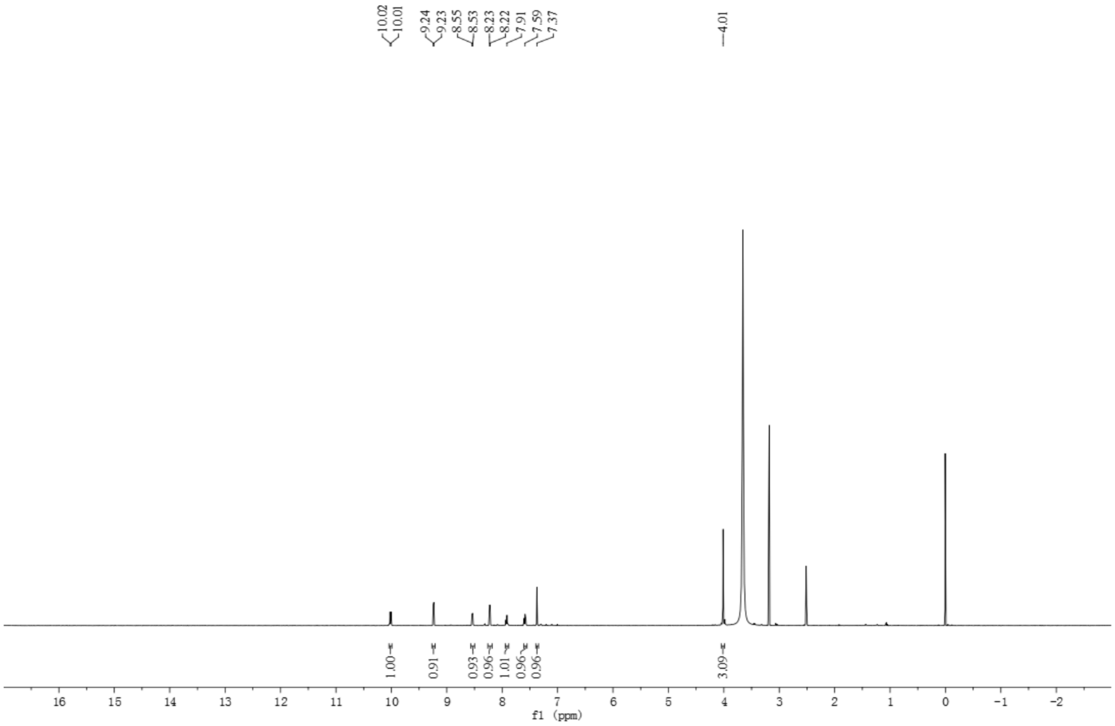

Supplementary Figure 23: ^{1}H NMR (600MHz, DMSO-d_6) of complex 2.

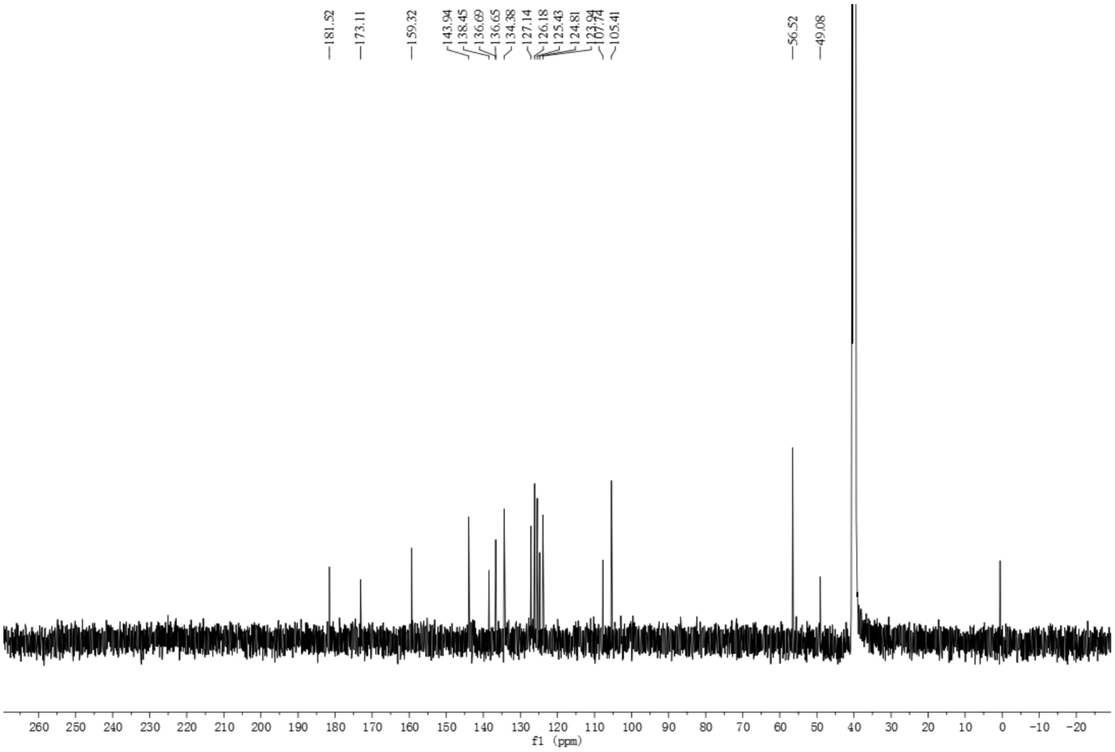

Supplementary Figure 24: ^{13}C NMR (600MHz, DMSO-d_6) of complex 2.

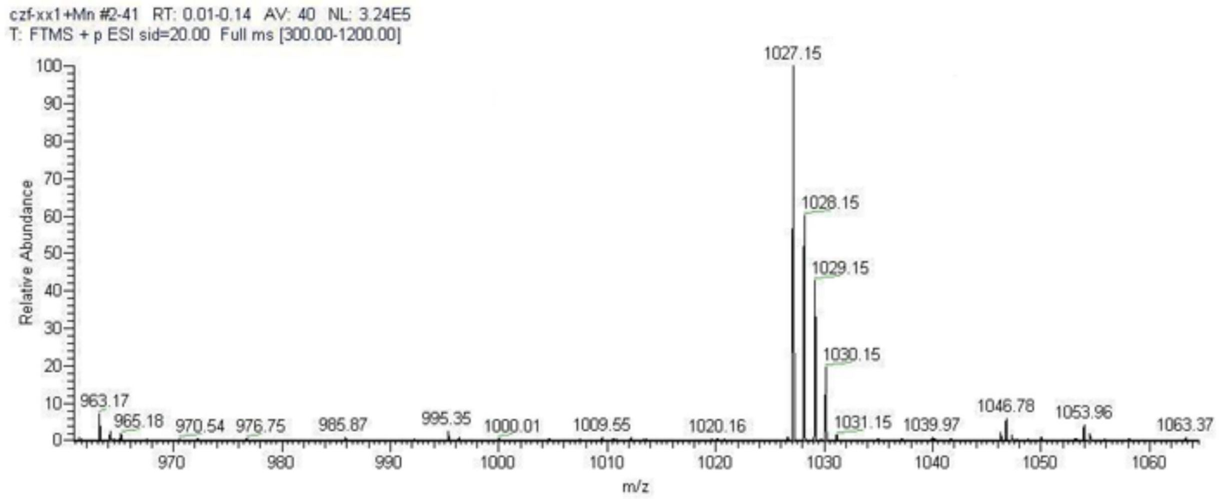

Supplementary Figure 25: ESI-MS analysis of complex 3.

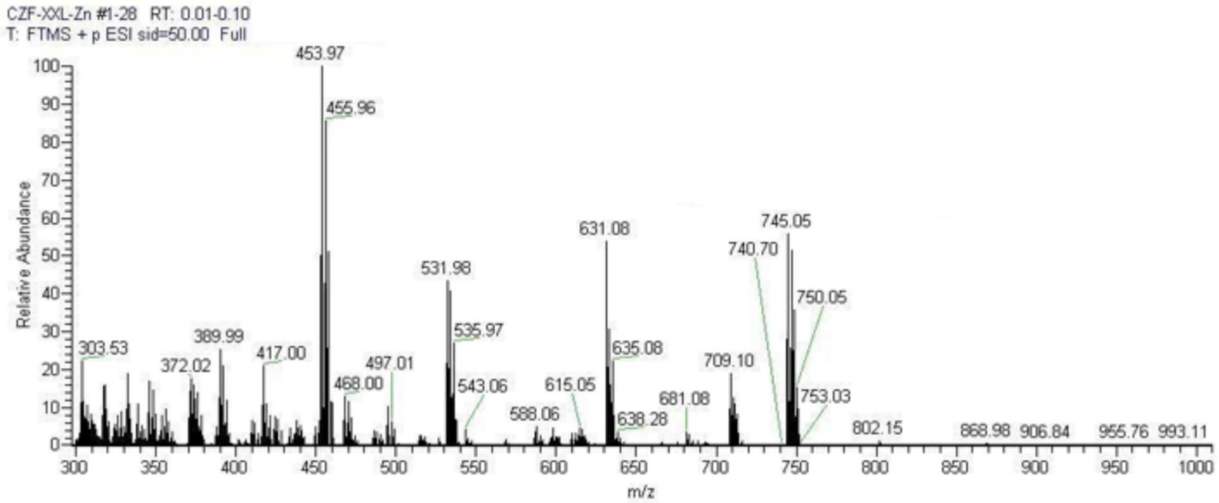

Supplementary Figure 26: ESI-MS analysis of complex 4.

Supplementary Table 1: Crystallographic data and refinements of I, III, IV and LY

	I	III	IV	LY
Formula	$C_{18}H_{20}BrNO_3$	$C_{20}H_{20}BrNO_4$	$C_{20}H_{21}NO_4$	$C_{18}H_{13}NO_3$
M_r	378.06	417.06	339.15	291.09
Crystal system	*Monoclinic*	*Triclinic*	*Triclinic*	*Monoclinic*
Space group	*P2(1)/c*	*P-1*	*P-1*	*P2(1)/c*
a/Å	4.9596(2)	7.5967(13)	6.128(7)	3.9612(7)
b/Å	11.0609(4)	11.669(2)	16.047(19)	17.756(7)
c/Å	31.5408(8)	11.784(2)	16.380(19)	18.899(3)
a/°	90.00	107.649(2)	89.954(13)	90.00
β/°	96.838(3)	100.701(3)	89.992(13)	90.00
γ/°	90.00	103.88(2)	89.968(13)	90.00
V/Å^3	1717.95(10)	2283.28 (10)	16116(3)	1329.3(6)
T/K	293(2)	296(2)	296(2)	293(2)
Z	4	2	1	4
D_c/g cm^{-3}	1.462	1.497	1.399	1.32
θ/°	6.38 to 52.74	3.78 to 50.7	2.48 to 50.24	6.86 to 52.72
F(000)	776	428	720	528
μ(Mo Ka)/mm^{-1}	2.407	2.240	0.098	0.076
Total no. reflns	17154	7336	8194	5311
No. indep. reflns	3504	3379	7653	2622
R1 [$I > 2\sigma(I)$]	0.1045	0.0354	0.0820	0.0530
$wR2$(all data)	0.1700	0.1012	0.1713	0.1301
Gof(F^2)	4.395	1.136	0.950	1.053

Supplementary Table 2: Selected bond lengths[Å] and angles [°]of I, III, IVand LY.

See Supplementary File 1

Supplementary Table 3: Crystallographic data and refinements of 1–4

	**1**	**2**	**3**	**4**
Formula	$C_{22}H_{31}Cl_2NO_8RuS_2$	$C_{18}H_{15}Cl_3RhNO_5$	$C_{56}H_{41}Cl_8MnN_3O_{17}$	$C_{36}H_{26}Cl_2ZnN_2O_{14}$
M_r	672.99	532.90	1361.9	844.0
Crystal system	*Monoclinic*	*Monoclinic*	*Triclinic*	Triclinic
Space group	*P2(1)/c*	*P2(1)/n*	*P-1*	*P-1*
a/Å	18.7733(11)	14.9430(12)	12.7355(19)	9.8420(11)
b/Å	13.0620(7)	6.2377(6)	.378(2)	.8601(15)
c/Å	11.8882(11)	20.6514(19)	17.839(2)	10.6253(14)
a/°	90.00	90.00	112.096(13)	114.573(14)
β/°	107.06(8)	96.192(8)	98.476(11)	96.185(10)
γ/°	90.00	90.00(2)	106.104(13)	93.337(11)
$V/Å^3$	2786.9(3)	2283.28 (10)	2983.3(7)	832.1(2)
T/K	293(2)	293(2)	293(2)	293(2)
Z	4	4	2	1
D_c/g cm^{-3}	1.605	1.855	1.521	1.638
θ/°	5.8 to 52.74	6.28 to 52.74	5.66 to 50.04	5.84 to 52.72
F(000)	1376	1064	1390	406
μ(Mo K*a*)/mm^{-1}	0.949	1.342	0.652	0.976
Total no. reflns	21065	10256	20965	6923
No. indep. reflns	5690	3903	10424	3390
R1 [$I > 2\sigma(I)$]	0.0793	0.0684	0.1074	0.0582
*wR*2(all data)	0.2502	0.1942	0.3399	0.1373
Gof(F^2)	1.054	1.059	0.999	1.039

Supplementary Table 4: Selected bond lengths[Å] and angles [°]of complexes 1–4

Complex **1**					
Ru(1)-Cl(1)	2.396(2)	Ru(1)-S(1)	2.2584(19)	Ru(1)-O(1)	2.102(6)
Ru(1)-Cl(2)	2.395(2)	Ru(1)-S(2)	2.231(2)	Ru(1)-N(1)	2.448(4)
S(1)-Ru(1)-Cl(1)	95.88(8)	N(1)-Ru(1)-Cl(1)	86.95(17)	O(1)-Ru(1)-Cl(2)	91.04(15)
S(1)-Ru(1)-Cl(2)	89.92(8)	N(1)-Ru(1)-Cl(2)	86.82(17)	O(1)-Ru(1)-S(1)	89.07(12)
S(2)-Ru(1)-Cl(1)	90.61(9)	N(1)-Ru(1)-S(1)	166.41(19)	O(1)-Ru(1)-S(2)	176.27(14)
S(2)-Ru(1)-Cl(2)	91.62(8)	Cl(1)-Ru(1)-Cl(2)	173.65(7)	O(1)-Ru(1)-N(1)	77.8(2)
S(1)-Ru(1)-S(2)	93.54(9)	O(1)-Ru(1)-Cl(1)	86.48(16)	N(1)-Ru(1)-S(2)	99.73(18)
Complex **2**					
Rh(1)-Cl(1)	2.334(3)	Rh(1)-Cl(3)	2.347(3)	Rh(1)-O(4)	2.066(6)
Rh(1)-Cl(2)	2.316(2)	Rh(1)-O(1)	2.038(5)	Rh(1)-N(1)	1.973(5)
Cl(1)-Rh(1)-Cl(2)	91.43(10)	O(1)-Rh(1)-Cl(2)	90.5(3)	O(1)-Rh(1)-Cl(3)	90.53(18)
Cl(1)-Rh(1)-Cl(3)	177.11(8)	O(1)-Rh(1)-Cl(3)	87.1(3)	N(1)-Rh(1)-Cl(1)	89.00(17)
Cl(2)-Rh(1)-Cl(3)	90.97(9)	N(4)-Rh(1)-O(4)	171.8(4)	N(1)-Rh(1)-Cl(2)	96.86(16)
O(1)-Rh(1)-O(4)	90.7(3)	O(1)-Rh(1)-Cl(1)	87.05(18)	N(1)-Rh(1)-Cl(3)	89.12(17)
O(4)-Rh(1)-Cl(1)	94.5(3)	O(1)-Rh(1)-Cl(2)	278.16(17)	N(4)-Rh(1)-O(1)	82.08(19)
Complex **3**					
Mn(1)-O(1)	2.242(6)	N(3)-Mn(1)-O(4)	96.3(3)	N(1)-Mn(1)-O(1)	73.6(2)
Mn(1)-O(4)	2.212(6)	N(3)-Mn(1)-O(7)	74.3(3)	N(1)-Mn(1)-O(4)	104.7(3)
Mn(1)-O(7)	2.195(6)	N(1)-Mn(1)-N(2)	102.5(3)	N(1)-Mn(1)-O(7)	95.6(2)
O(1)-Mn(1)-O(4)	78.7(2)	N(1)-Mn(1)-N(3)	146.2(3)	N(2)-Mn(1)-O(1)	149.6(3)
O(1)-Mn(1)-O(7)	123.7(2)	Mn(1)-N(1)	2.170(7)	N(2)-Mn(1)-O(4)	73.2(3)
O(4)-Mn(1)-O(7)	153.8(3)	Mn(1)-N(2)	2.229(8)	N(2)-Mn(1)-O(7)	86.5(3)
N(3)-Mn(1)-O(1)	85.3(3)	Mn(1)-N(3)	2.167(8)	N(2)-Mn(1)-N(3)	108.8(3)
Complex **4**					
Zn(1)-O(1)	2.114(2)	N(1)-Zn(1)-O(4)	92.22(12)	O(1)^{1}-Zn(1)-O(4)	94.86(10)
Zn(1)-O(4)	2.241(3)	N(1)-Zn(1)-O(1)^{1}	100.00(11)	O(1)^{1}-Zn(1)-O(4)^{1}	85.14(10)
Zn(1)-N(1)	2.023(3)	N(1)-Zn(1)-O(4)^{1}	87.78(12)	O(4)-Zn(1)-O(4)^{1}	180.00(15)
O(1)-Zn(1)-O(1)^{1}	180.00(14)	N(1)-Zn(1)-N(1)^{1}	180.00(13)	N(1)^{1}-Zn(1)-O(1)	100.00(11)
O(1)-Zn(1)-O(4)	85.14(10)	Zn(1)-O(1)^{1}	2.114(2)	N(1)^{1}-Zn(1)-O(4)	87.78(12)
O(1)-Zn(1)-O(4)^{1}	94.86(10)	Zn(1)-O(2)^{1}	2.241(3)	N(1)^{1}-Zn(1)-O(1)^{1}	80.00(11)
N(1)-Zn(1)-O(1)	80.00(11)	Zn(1)-N(1)^{1}	2.023(3)	N(1)^{1}-Zn(1)-N(1)^{1}	92.22(12)

Supplementary Table 5: The inhibitive ratios (%) of LY, 1–4 and metal salt towards four cancer-cell lines and one normal liver cell line HL-7702 for 48 h

	BEL-7404	Hep-G2	NCI-H460	T-24	HL-7702
**LY**	32.27±1.73	53.13±1.19	52.85±0.32	42.40±2.14	45.49±0.78
**1**	31.32±1.32	18.83±0.56	48.03±3.51	50.41±3.18	41.23±2.38
**2**	37.14±2.55	74.94±0.95	63.82±3.26	49.62±1.61	15.11±0.91
**3**	35.27±1.15	62.99±0.43	54.93±0.86	45.42±1.66	32.97±1.14
**4**	37.41±1.53	20.39±0.96	37.84±1.53	53.81±2.39	38.03±1.67
$RuCl_3 \cdot 3H_2O$	32.81±1.52	25.01±0.99	27.71±0.63	30.23±0.78	29.21±0.99
$RhCl_3 \cdot 3H_2O$	35.29±0.97	24.02±1.02	25.41±1.25	27.09±1.11	30.12±1.57
$Mn(ClO_4)_2 \cdot 6H_2O$	25.84±1.36	18.52±0.67	30.12±0.46	24.08±1.86	23.06±1.64
$Zn(ClO_4)_2 \cdot 6H_2O$	29.55±1.09	20.18±0.79	24.08±0.59	20.16±1.09	22.34±1.47
**cisplatin**	55.15±1.18	60.63±0.99	53.88±1.29	47.58±2.05	68.95±1.42

Results represent mean ± SD of at least five independent experiments. SD represents the standard deviation. The concentration of **1–4** and LY are 20 μmol/L, the corresponding salts were 1×10^{-4} mol/L, cisplatin was dissolved at a concentration of 1mM in 0.154 M NaCl.

Supplementary Table 6: Lists of changes in relative expression for cell cycle regulators genes in the Hep-G2 cells after treated with 2 (7 μM) for 24 h. The table lists genes that exhibit a difference in expression in the Hep-G2 cells sample when compared to control.

Supplementary Table 7: Lists of changes in relative expression for cell cycle regulators genes in the Hep-G2 cells after treated with 3 (14 μM) for 24 h. The table lists genes that exhibit a difference in expression in the Hep-G2 cells sample when compared to control.

Supplementary Table 8: Lists of changes in relative expression for apoptosis genes in the Hep-G2 cells after treated with 2 (7 μM) for 24 h. The table lists genes that exhibit a difference in expression in the Hep-G2 cells sample when compared to control.

Supplementary Table 9: Lists of changes in relative expression for apoptosis genes in the Hep-G2 cells after treated with 3 (14 μM) for 24 h. The table lists genes that exhibit a difference in expression in the Hep-G2 cells sample when compared to control.

See Supplementary File 1
