# Supplementary material for: Facile total synthesis of lysicamine and the anticancer activities of the RuII, RhIII, MnII and ZnII complexes of lysicamine
Source: Oncotarget. 2017 Jul 26;8(35):59359–75. doi: 10.18632/oncotarget.19584 (PMC5601738; doi:10.18632/oncotarget.19584)
Supplement: Supplementary file 2 [file oncotarget-08-59359-s002.docx]

**Supplementary Table 2: Selected bond lengths[Å] and angles [°]of I, III, IVand LY**

| \| **Compound** (**I**) \| \| \| \| \| \| \| \| \| \| \| \| --- \| --- \| --- \| --- \| --- \| --- \| --- \| --- \| --- \| --- \| --- \| \| Br(1)−C(1) \| 1.904(7) \| \| C(1)−C(2) \| \| 1.365(7) \| \| C(9)−C(10) \| \| 1.512(7) \| \| \| O(1)−C(8) \| 1.235(6) \| \| C(1)−C(6) \| \| 1.342(7) \| \| C(10)−C(11) \| \| 1.495(8) \| \| \| O(1)−C(17) \| 1.419(6) \| \| C(2)−C(3) \| \| 1.444(8) \| \| C(11)−C(12) \| \| 1.404(7) \| \| \| O(2)−C(13) \| 1.357(6) \| \| C(3)−C(4) \| \| 1.344(8) \| \| C(11)−C(16) \| \| 1.367(7) \| \| \| O(3)−C(14) \| 1.365(7) \| \| C(4)-C(5) \| \| 1.175(8) \| \| C(12)−C(13) \| \| 1.367(7) \| \| \| O(3)−O(18) \| 1.424(6) \| \| C(5)−C(6) \| \| 1.427(8) \| \| C(13)−C(14) \| \| 1.410(8) \| \| \| N(1)−C(8) \| 1.313(7) \| \| C(6)−C(7) \| \| 1.466(8) \| \| C(14)−C(15) \| \| 1.366(8) \| \| \| N(1)−C(9) \| 1.443(6) \| \| C(7)−C(8) \| \| 1.507(8) \| \| C(15)−C(16) \| \| 1.388(8) \| \| \| C(13)−O(2)−C(17) \| \| 116.5(5) \| \| C(1)−C(6)−C(5) \| \| 113.4(7) \| \| C(16)−C(11)−C(10) \| \| 120.8(6) \| \| C(14)−O(3)−C(18) \| \| 117.5(5) \| \| C(1)−C(6)−C(7) \| \| 128.9(7) \| \| C(16)−C(11)−C(12) \| \| 117.5(6) \| \| C(8)−N(1)−C(9) \| \| 124.4(5) \| \| C(5)−C(6)−C(7) \| \| 117.7(7) \| \| C(13)−C(12)−C(11) \| \| 122.4(6) \| \| (2)−C(1)−Br(1) \| \| 116.3(6) \| \| C(6)−C(7)−C(8) \| \| 115.7(5) \| \| O(2)−C(13)−C(12) \| \| 125.8(5) \| \| C(6)−C(1)−Br(1) \| \| 118.5(6) \| \| O(1)−C(8)−N(1) \| \| 122.9(6) \| \| O(2)−C(13)−C(14) \| \| 115.3(6) \| \| C(6)−C(1)−C(2) \| \| 125.2(7) \| \| O(1)−C(8)−C(7) \| \| 122.3(6) \| \| C(12)−C(13)−C(14) \| \| 118.9(6) \| \| C(1)−C(2)−C(3) \| \| 112.9(7) \| \| N(1)−C(8)−C(7) \| \| 114.8(5) \| \| O(3)−C(14)−C(13) \| \| 116.1(6) \| \| C(4)−C(3)−C(2) \| \| 121.2(7) \| \| N(1)－C(9)−C(10) \| \| 112.7(5) \| \| O(3)−C(14)−C(15) \| \| 124.9(6) \| \| C(5)−C(4)−C(3) \| \| 120.7(7) \| \| C(11)−C(10)−C(9) \| \| 114.3(5) \| \| C(15)−C(14)−C(13) \| \| 119.1(6) \| \| C(4)−C(5)−C(6) \| \| 126.5(8) \| \| C(12)−C(11)−C(10) \| \| 121.6(5) \| \|  \| \|  \| \| Compound **(III)** \| \|  \| \|  \| \|  \| \|  \| \|  \| | | | | | |
| --- | --- | --- | --- | --- | --- | --- | --- | --- | --- | --- | --- | --- | --- | --- | --- | --- | --- | --- | --- | --- | --- | --- | --- | --- | --- | --- | --- | --- | --- | --- | --- | --- | --- | --- | --- | --- | --- | --- | --- | --- | --- | --- | --- | --- | --- | --- | --- | --- | --- | --- | --- | --- | --- | --- | --- | --- | --- | --- | --- | --- | --- | --- | --- | --- | --- | --- | --- | --- | --- | --- | --- | --- | --- | --- | --- | --- | --- | --- | --- | --- | --- | --- | --- | --- | --- | --- | --- | --- | --- | --- | --- | --- | --- | --- | --- | --- | --- | --- | --- | --- | --- | --- | --- | --- | --- | --- | --- | --- | --- | --- | --- | --- | --- | --- | --- | --- | --- | --- | --- | --- | --- | --- | --- | --- | --- | --- | --- | --- | --- | --- | --- | --- | --- | --- | --- | --- | --- | --- | --- | --- | --- | --- | --- | --- | --- | --- | --- | --- | --- | --- | --- | --- | --- | --- | --- | --- | --- | --- | --- | --- | --- | --- | --- | --- | --- | --- | --- | --- | --- | --- | --- | --- | --- | --- | --- | --- | --- | --- | --- | --- | --- | --- | --- | --- | --- | --- | --- | --- | --- | --- | --- | --- | --- | --- | --- | --- | --- | --- | --- | --- | --- | --- | --- | --- | --- | --- | --- | --- | --- | --- | --- | --- | --- | --- | --- | --- | --- | --- | --- | --- | --- | --- | --- | --- | --- |
| C(13)−O(2)−C(14) | 117.8(3) | C(1)－C(6)−C(5) | 116.9(2) | O(1)−C(11)−C(13) | 115.3(2) |
| C(19)−O(4)−C(20) | 116.6(2) | O(1)−C(6)−C(7) | 121.9(2) | O(10)−C(11)−C(13) | 119.4(2) |
| C(8)−N(1)−C(18) | 113.6(2) | O(5)−C(6)−C(7) | 121.2(2) | O(2)−C(13)−C(11) | 114.5(2) |
| C(19)−N(1)−C(8) | 123.2(2) | C(8)−C(7)−C(6) | 125.7(2) | C(15)−C(13)−O(2) | 126.1(2) |
| C(19)−N(1)−C(18) | 119.1(2) | N(1)−C(8)−C(9) | 114.00(18) | C(15)−C(13)−C(11) | 119.4(2) |
| C(2)−C(1)−Br(1) | 118.74(18) | C(7)−C(8)−N(1) | 120.5(2) | C(13)−C(15)−C(16) | 122.0(2) |
| C(2)−C(1)−C(6) | 122.4(2) | C(7)−C(8)−C(9) | 125.4(2) | C(9)−C(16)−C(15) | 119.1(2) |
| C(6)−C(1)−Br(1) | 118.80(16) | C(10)−C(9)−C(8) | 121.05(19) | C(9)−C(16)−C(17) | 121.0(2) |
| C(3)−C(2)−C(1) | 119.0(2) | C(16)−C(9)−C(8) | 120.6(2) | C(15)−C(16)−C(17) | 119.8(2) |
| C(4)−C(3)−C(2) | 120.0(2) | C(16)−C(9)−C(10) | 118.4(2) | C(16)−C(17)−C(18) | 111.7(2) |
| C(3)−C(4)−C(5) | 120.3(2) | C(11)−C(10)−C(9) | 121.8(2) | N(1)−C(18)−C(17) | 108.2(2) |
| C(4)−C(5)－C(6) | 121.3(2) | O(1)−C(11)−C(10) | 125.4(2) | O(3)−C(19)−O(4) | 124.5(2) |
| Compound (**IV**) |  |  |  |  |  |
| O(1)−C(1) | 1.428(14) | C(2)−C(3) | 1.361(18) | C(11)−C(16) | 1.407(17) |
| O(1)−C(2) | 1.380(16) | C(2)−C(19) | 1.370(16) | C(12)−C(13) | 1.360(17) |
| O(2)−C(19) | 1.389(14) | C(3)−C(4) | 1.389(17) | C(13)−C(14) | 1.368(18) |
| O(2)−C(20) | 1.424(14) | C(4)−C(5) | 1.521(17) | C(14)−C(15) | 1.395(16) |
| O(3)−C(7) | 1.168(14) | C(4)−C(18) | 1.380(15) | C(15)−C(16) | 1.388(16) |
| O(4)−C(7) | 1.344(15) | C(5)−C(6) | 1.514(17) | C(16)−C(17) | 1.471(16) |
| O(4)−C(8) | 1.449(16) | C(9)−C(10) | 1.490(15) | C(17)−C(18) | 1.394(16) |
| N(1)−C(6) | 1.424(13) | C(9)−C(18) | 1.487(16) | C(17)−C(19) | 1.353(16) |
| N(1)−C(7) | 1.366(16) | C(10)−C(11) | 1.460(17) |  |  |
| N(1)−C(9) | 1.444(15) | C(11)−C(12) | 1.379(17) |  |  |
| C(1)−O(1)−C(2) | 113.0(11) | C(6)−C(5)−C(4) | 109.1(11) | O(12)−C(13)−C(14) | 118.9(13) |
| C(19)−O(2−C(20) | 114.1(9) | N(1)−C(6)−C(5) | 111.1(10) | C(13)−C(14)−C(15) | 120.8(13) |
| C(7)−O(4)−C(8) | 114.7(10) | O(3)−C(7)−O(4) | 124.2(14) | C(16)−C(15)−C(14) | 120.4(13) |
| C(6)−N(1)−C(9) | 118.8(11) | O(3)−C(7)−N(1) | 111.6(12) | C(15)−C(16)−C(17) | 122.8(11) |
| C(6)−N(1)−C(7) | 123.5(12) | O(4)−C(7)−N(1) | 111.6(12) | C(18)−C(17)−C(16) | 116.6(11) |
| C(7)−N(1)−C(9) | 117.2(10) | N(1)−C(9)−C(10) | 113.2(10) | C(19)−C(17)−C(16) | 125.7(12) |
| C(3)−C(2)−O(1) | 127.0(12) | N(1)−C(9)−C(18) | 113.4(10) | C(19)−C(17)−C(18) | 117.6(11) |
| C(3)−C(2)−C(19) | 118.8(13) | C(18)−C(9)−C(10) | 109.1(10) | C(4)−C(18)−C(9) | 121.5(11) |
| C(19)−C(2)−O(1) | 114.1(12) | C(11)−C(10)−C(9) | 110.2(10) | C(4)−C(18)−C(17) | 120.6(12) |
| C(2)−C(3)−C(4) | 120.3(13) | C(12)−C(11)−C(10) | 122.0(13) | C(17)−C(18)−C(9) | 117.9(10) |
| C(3)−C(4)−C(5) | 118.7(12) | O(12)−C(11)−C(16) | 119.5(12) | C(2)−C(19)−O(2) | 117.0(12) |
| C(18)−C(4)−C(3) | 119.4(12) | O(16)−C(11)−C(10) | 118.3(11) | C(17)−C(19)−O(2) | 119.6(11) |
| C(18)−C(4)−C(5) | 121.6(12) | O(13)−C(12)−C(11) | 122.2(13) | C(17)−C(19)−C(2) | 123.3(13) |
| **LY** |  |  |  |  |  |
| O(1)−C(2) | 1.220(3) | C(1)−C(18) | 1.408(3) | C(8)−C(9) | 1.483(3) |
| O(2)−C(10) | 1.367(2) | C(2)−C(3) | 1.467(3) | C(9)−C(10) | 1.379(3) |
| O(2)−C(11) | 1.446(2) | C(3)−C(4) | 1.396(3) | C(9)−C(18) | 1.435(3) |
| O(3)−C(12) | 1.348(2) | C(3)−C(8) | 1.404(3) | C(10)−C(12) | 1.430(3) |
| O(3)−C(13) | 1.432(2) | C(4)−C(5) | 1.362(4) | C(12)−C(14) | 1.355(3) |
| N(1)−C(1) | 1.329(3) | C(5)−C(6) | 1.372(4) | C(14)−C(15) | 1.407(3) |
| N(1)−C(17) | 1.340(3) | C(6)−C(7) | 1.377(3) | C(15)−C(16) | 1.397(3) |
| C(1)−C(2) | 1.480(3) | C(7)−C(8) | 1.400(3) | C(15)−C(18) | 1.417(3) |
| C(10)−O(2)−C(11) | 115.48(15) | C(4)−C(5)−C(6) | 118.4(2) | C(16)−C(17) | 1.360(3) |
| C(12)−O(3)−C(13) | 117.30(15) | C(5)−C(6)−C(7) | 121.7(3) | O(3)−C(12)−C(14) | 124.64(19) |
| C(1)−N(1)−C(17) | 116.8(2) | C(6)−C(7)−C(8) | 121.2(2) | C(14)−C(12)−C(10) | 120.88(18) |
| N(1)−C(1)−C(2) | 115.7(2) | C(3)−C(8)−C(9) | 119.9(2) | O(12)−C(14)−C(15) | 118.9(2) |
| N(1)−C(1)−C(18) | 124.1(2) | C(7)−C(8)−C(3) | 116.6(2) | O(14)−C(15)−C(18) | 120.95(18) |
| C(18)−C(1)−C(2) | 120.1(2) | C(7)−C(8)−C(9) | 123.49(19) | O(16)−C(15)−C(14) | 121.4(2) |
| O(1)−C(2)−C(1) | 121.8(2) | C(10)−C(9)−C(8) | 125.07(18) | C(16)−C(15)−C(18) | 117.7(2) |
| O(1)−C(2)−C(3) | 120.9(2) | C(10)−C(9)−C(18) | 116.73(18) | C(17)−C(16)−C(15) | 119.4(2) |
| C(3)−C(2)−C(1) | 117.38(19) | C(18)−C(9)−C(8) | 118.21(18) | N(11)−C(17)−C(16) | 124.6(2) |
| C(4)−C(3)−C(2) | 117.5(2) | O(2)−C(10)−C(9) | 121.93(18) | C(1)−C(18)−C(9) | 122.4(2) |
| C(2)−C(3)−C(8) | 120.6(2) | O(2)−C(10)−C(12) | 115.63(17) | C(1)−C(18)−C(15) | 117.35(14) |
| C(8)−C(3)−C(2) | 121.9(2) | C(9)−C(10)−C(12) | 122.34(18) | C(15)−C(18)−C(9) | 120.23(17) |
| C(5)−C(4)−C(3) | 121.5(3) | O(3)−C(12)−C(10) | 114.48(17) |  |  |

**Supplementary Table 6: Lists of changes in relative expression for cell cycle regulators genes in the Hep-G2 cells after treated with 2 (7 μM) for 24 h. The table lists genes that exhibit a difference in expression in the Hep-G2 cells sample when compared to control.**

| **Gene** | **Fold Change** | ***p* value** | **Fold up/ down-regulation** | **Comments** |
| --- | --- | --- | --- | --- |
| ABL1 | 0.54 | **0.008406** | -1.85 | OKAY |
| ANAPC2 | 0.71 | 0.408460 | -1.42 | OKAY |
| ATM | **0.29** | **0.030197** | **-3.45** | OKAY |
| ATR | **0.43** | **0.015783** | **-2.32** | OKAY |
| AURKA | 0.51 | **0.003687** | -1.95 | OKAY |
| AURKB | **0.46** | **0.000240** | **-2.19** | OKAY |
| BCCIP | 1.40 | **0.029125** | 1.40 | OKAY |
| BCL2 | 0.81 | 0.257688 | -1.23 | OKAY |
| BIRC5 | **0.41** | **0.000073** | **-2.45** | OKAY |
| BRCA1 | 0.54 | **0.008402** | -1.87 | OKAY |
| BRCA2 | 0.52 | 0.093541 | -1.94 | OKAY |
| CASP3 | 1.81 | **0.014490** | 1.81 | OKAY |
| CCNA2 | **0.38** | **0.000075** | **-2.61** | OKAY |
| CCNB1 | 0.55 | **0.002101** | -1.80 | OKAY |
| CCNB2 | **0.37** | **0.000511** | **-2.73** | OKAY |
| CCNC | 1.23 | 0.135078 | 1.23 | OKAY |
| CCND1 | **0.50** | **0.001591** | **-2.01** | OKAY |
| CCND2 | **3.59** | 0.081054 | **3.59** | A |
| CCND3 | 1.17 | 0.317202 | 1.17 | OKAY |
| CCNE1 | **5.66** | **0.000389** | **5.66** | OKAY |
| CCNF | **0.18** | **0.000013** | **-5.55** | OKAY |
| CCNG1 | **0.30** | **0.001056** | **-3.29** | OKAY |
| CCNG2 | **2.36** | **0.000280** | **2.36** | OKAY |
| CCNH | 1.28 | 0.125620 | 1.28 | OKAY |
| CCNT1 | 1.79 | 0.059991 | 1.79 | OKAY |
| CDC16 | 0.70 | 0.079894 | -1.43 | OKAY |
| CDC20 | 0.73 | **0.022479** | -1.36 | OKAY |
| CDC25A | **2.08** | **0.011288** | **2.08** | OKAY |
| CDC25C | 0.70 | **0.003459** | -1.43 | OKAY |
| CDC34 | 1.95 | 0.102403 | 1.95 | OKAY |
| CDC6 | 1.64 | **0.010953** | 1.64 | OKAY |
| CDK1 | 0.59 | **0.009334** | -1.70 | OKAY |
| CDK2 | **0.45** | **0.000067** | **-2.21** | OKAY |
| CDK4 | **0.47** | **0.000688** | **-2.14** | OKAY |
| CDK5R1 | **10.92** | **0.000028** | **10.92** | A |
| CDK5RAP1 | 1.52 | 0.205094 | 1.52 | OKAY |
| CDK6 | **0.33** | **0.000218** | **-3.07** | OKAY |
| CDK7 | **2.44** | **0.012412** | **2.44** | OKAY |
| CDK8 | 1.17 | 0.396472 | 1.17 | OKAY |
| CDKN1A | **8.89** | **0.000742** | **8.89** | OKAY |
| CDKN1B | **2.38** | **0.017427** | **2.38** | OKAY |
| CDKN2A | 0.74 | **0.036235** | -1.36 | OKAY |
| CDKN2B | 1.02 | 0.991271 | 1.02 | OKAY |
| CDKN3 | 0.76 | 0.077155 | -1.31 | OKAY |
| CHEK1 | 0.66 | **0.045255** | -1.51 | OKAY |
| CHEK2 | **0.44** | **0.004382** | **-2.30** | OKAY |
| CKS1B | 0.73 | **0.011680** | -1.37 | OKAY |
| CKS2 | 1.22 | **0.040725** | 1.22 | OKAY |
| CUL1 | 0.59 | **0.030568** | -1.70 | OKAY |
| CUL2 | 0.84 | 0.208812 | -1.19 | OKAY |
| CUL3 | 0.74 | 0.080003 | -1.35 | OKAY |
| E2F1 | 0.76 | 0.307596 | -1.32 | OKAY |
| E2F4 | 0.92 | 0.720460 | -1.09 | OKAY |
| GADD45A | **22.72** | **0.019096** | **22.72** | OKAY |
| GTSE1 | **0.27** | **0.000048** | **-3.74** | OKAY |
| HUS1 | 0.62 | **0.019045** | -1.60 | OKAY |
| KNTC1 | **0.41** | **0.008915** | **-2.44** | OKAY |
| KPNA2 | 0.88 | 0.226955 | -1.13 | OKAY |
| MAD2L1 | **0.45** | **0.000726** | **-2.23** | OKAY |
| MAD2L2 | 0.92 | 0.334832 | -1.09 | OKAY |
| MCM2 | **0.37** | **0.003075** | **-2.67** | OKAY |
| MCM3 | 0.60 | **0.010760** | -1.66 | OKAY |
| MCM4 | **0.46** | **0.001045** | **-2.15** | OKAY |
| MCM5 | 0.82 | **0.048197** | -1.22 | OKAY |
| MDM2 | 1.21 | 0.212047 | 1.21 | OKAY |
| MKI67 | **0.32** | **0.000282** | **-3.15** | OKAY |
| MNAT1 | 1.21 | 0.232595 | 1.21 | OKAY |
| MRE11A | **0.50** | **0.028192** | **-2.02** | OKAY |
| NBN | **0.48** | **0.012327** | **-2.07** | OKAY |
| RAD1 | 0.85 | 0.335501 | -1.17 | OKAY |
| RAD17 | 0.50 | **0.019271** | -1.99 | OKAY |
| RAD51 | 0.52 | **0.021926** | -1.92 | OKAY |
| RAD9A | **2.53** | **0.006775** | **2.53** | OKAY |
| RB1 | **0.43** | **0.003792** | **-2.35** | OKAY |
| RBBP8 | 0.67 | **0.020003** | -1.50 | OKAY |
| RBL1 | **0.41** | **0.002688** | **-2.46** | OKAY |
| RBL2 | **0.33** | **0.000893** | **-3.00** | OKAY |
| SERTAD1 | **15.48** | **0.000642** | **15.48** | OKAY |
| SKP2 | **0.25** | **0.000020** | **-3.98** | OKAY |
| STMN1 | 0.52 | **0.002598** | -1.93 | OKAY |
| TFDP1 | **0.14** | **0.000624** | **-7.15** | OKAY |
| TFDP2 | 0.73 | **0.041699** | -1.37 | OKAY |
| TP53 | 0.60 | **0.000512** | -1.66 | OKAY |
| WEE1 | 1.19 | 0.159900 | 1.19 | OKAY |
| ACTB | **0.45** | **0.001167** | **-2.23** | OKAY |
| B2M | 0.67 | **0.047538** | -1.49 | OKAY |
| GAPDH | 0.95 | 0.327416 | -1.05 | OKAY |
| HPRT1 | **0.46** | **0.001103** | **-2.15** | OKAY |
| RPLP0 | 1.05 | 0.330837 | 1.05 | OKAY |

**Supplementary Table 7: Lists of changes in relative expression for cell cycle regulators genes in the Hep-G2 cells after treated with 3 (14 μM) for 24 h. The table lists genes that exhibit a difference in expression in the Hep-G2 cells sample when compared to control.**

| **Symbol** | **Fold Change** | **p value** | **Fold up/ down-regulation** | **Comments** |
| --- | --- | --- | --- | --- |
| ABL1 | 1.11 | 0.090575 | 1.11 | OKAY |
| ANAPC2 | 1.38 | 0.208963 | 1.38 | OKAY |
| ATM | 1.13 | 0.752241 | 1.13 | OKAY |
| ATR | 1.14 | 0.453764 | 1.14 | OKAY |
| AURKA | 0.71 | **0.016821** | -1.41 | OKAY |
| AURKB | 0.96 | 0.168863 | -1.04 | OKAY |
| BCCIP | 1.19 | 0.107606 | 1.19 | OKAY |
| BCL2 | 1.32 | 0.187829 | 1.32 | OKAY |
| BIRC5 | 0.74 | **0.009937** | -1.36 | OKAY |
| BRCA1 | 1.24 | 0.079818 | 1.24 | OKAY |
| BRCA2 | **2.15** | **0.012414** | **2.15** | OKAY |
| CASP3 | 1.49 | **0.011811** | 1.49 | OKAY |
| CCNA2 | **0.39** | **0.000093** | **-2.58** | OKAY |
| CCNB1 | 0.78 | **0.031338** | -1.28 | OKAY |
| CCNB2 | 1.09 | 0.334035 | 1.09 | OKAY |
| CCNC | 1.57 | **0.005016** | 1.57 | OKAY |
| CCND1 | 0.71 | 0.140632 | -1.42 | OKAY |
| CCND2 | 1.06 | 0.728440 | 1.06 | B |
| CCND3 | 0.87 | 0.089910 | -1.14 | OKAY |
| CCNE1 | 1.16 | 0.193022 | 1.16 | OKAY |
| CCNF | **0.25** | **0.000027** | **-4.01** | OKAY |
| CCNG1 | 0.89 | 0.193171 | -1.13 | OKAY |
| CCNG2 | **2.49** | **0.001239** | **2.49** | OKAY |
| CCNH | 1.30 | 0.058695 | 1.30 | OKAY |
| CCNT1 | 1.51 | **0.032076** | 1.51 | OKAY |
| CDC16 | 0.89 | 0.319949 | -1.13 | OKAY |
| CDC20 | 0.98 | 0.854118 | -1.02 | OKAY |
| CDC25A | 0.64 | **0.004319** | -1.57 | OKAY |
| CDC25C | 0.83 | **0.021419** | -1.20 | OKAY |
| CDC34 | 1.96 | **0.022655** | 1.96 | OKAY |
| CDC6 | 1.48 | 0.081950 | 1.48 | OKAY |
| CDK1 | 0.99 | 0.861714 | -1.01 | OKAY |
| CDK2 | 0.57 | **0.000160** | -1.76 | OKAY |
| CDK4 | 1.04 | 0.436407 | 1.04 | OKAY |
| CDK5R1 | **2.71** | 0.123397 | **2.71** | B |
| CDK5RAP1 | **3.09** | **0.030044** | **3.09** | OKAY |
| CDK6 | **0.49** | **0.000186** | **-2.04** | OKAY |
| CDK7 | **2.50** | **0.005585** | **2.50** | OKAY |
| CDK8 | 1.19 | 0.126660 | 1.19 | OKAY |
| CDKN1A | **5.94** | **0.024508** | **5.94** | OKAY |
| CDKN1B | 1.66 | 0.131541 | 1.66 | OKAY |
| CDKN2A | 0.69 | **0.017611** | -1.46 | OKAY |
| CDKN2B | 0.91 | 0.553417 | -1.10 | OKAY |
| CDKN3 | 1.15 | 0.243117 | 1.15 | OKAY |
| CHEK1 | 1.22 | 0.165821 | 1.22 | OKAY |
| CHEK2 | 0.74 | 0.071619 | -1.35 | OKAY |
| CKS1B | 1.37 | **0.012627** | 1.37 | OKAY |
| CKS2 | 0.88 | **0.016655** | -1.14 | OKAY |
| CUL1 | 0.93 | 0.484375 | -1.07 | OKAY |
| CUL2 | 1.74 | **0.002245** | 1.74 | OKAY |
| CUL3 | 1.23 | **0.049588** | 1.23 | OKAY |
| E2F1 | 1.67 | 0.132218 | 1.67 | OKAY |
| E2F4 | 1.06 | 0.559084 | 1.06 | OKAY |
| GADD45A | **4.51** | **0.001233** | **4.51** | OKAY |
| GTSE1 | 0.87 | 0.143701 | -1.15 | OKAY |
| HUS1 | 0.88 | 0.301035 | -1.14 | OKAY |
| KNTC1 | 1.39 | 0.056059 | 1.39 | OKAY |
| KPNA2 | 0.75 | **0.037281** | -1.33 | OKAY |
| MAD2L1 | 0.97 | 0.612144 | -1.03 | OKAY |
| MAD2L2 | 1.71 | **0.000504** | 1.71 | OKAY |
| MCM2 | 0.75 | 0.066680 | -1.33 | OKAY |
| MCM3 | 1.12 | 0.283647 | 1.12 | OKAY |
| MCM4 | 0.77 | **0.047737** | -1.29 | OKAY |
| MCM5 | 0.75 | **0.024415** | -1.33 | OKAY |
| MDM2 | 1.50 | 0.121042 | 1.50 | OKAY |
| MKI67 | 0.60 | **0.002196** | -1.68 | OKAY |
| MNAT1 | **2.09** | **0.000793** | **2.09** | OKAY |
| MRE11A | 1.61 | **0.020917** | 1.61 | OKAY |
| NBN | 0.75 | 0.107477 | -1.34 | OKAY |
| RAD1 | 1.08 | 0.365008 | 1.08 | OKAY |
| RAD17 | 1.76 | **0.007422** | 1.76 | OKAY |
| RAD51 | 1.11 | 0.484556 | 1.11 | OKAY |
| RAD9A | 1.49 | **0.030817** | 1.49 | OKAY |
| RB1 | 1.08 | 0.423603 | 1.08 | OKAY |
| RBBP8 | 1.07 | 0.471734 | 1.07 | OKAY |
| RBL1 | 1.24 | 0.146748 | 1.24 | OKAY |
| RBL2 | 0.78 | 0.066528 | -1.28 | OKAY |
| SERTAD1 | **3.62** | **0.020426** | **3.62** | OKAY |
| SKP2 | **0.29** | **0.000135** | **-3.42** | OKAY |
| STMN1 | 0.56 | **0.003467** | -1.79 | OKAY |
| TFDP1 | **0.48** | **0.006486** | **-2.07** | OKAY |
| TFDP2 | 1.55 | **0.002725** | 1.55 | OKAY |
| TP53 | 0.92 | 0.636538 | -1.08 | OKAY |
| WEE1 | 1.12 | 0.457692 | 1.12 | OKAY |
| ACTB | **0.30** | **0.000294** | **-3.29** | OKAY |
| B2M | 1.29 | 0.104890 | 1.29 | OKAY |
| GAPDH | 0.91 | 0.064919 | -1.10 | OKAY |
| HPRT1 | 0.54 | **0.002561** | -1.87 | OKAY |
| RPLP0 | 1.10 | 0.067188 | 1.10 | OKAY |

**Supplementary Table 8: Lists of changes in relative expression for apoptosis genes in the Hep-G2 cells after treated with 2 (7 μM) for 24 h. The table lists genes that exhibit a difference in expression in the Hep-G2 cells sample when compared to control.**

| **Gene** | **Fold Change** | **p value** | **Fold up/ down-regulation** | **Comments** |
| --- | --- | --- | --- | --- |
| ABL1 | 0.61 | **0.002965** | -1.65 | OKAY |
| AIFM1 | 0.67 | **0.014279** | -1.50 | OKAY |
| AKT1 | **0.33** | **0.001956** | **-3.04** | OKAY |
| APAF1 | 1.44 | **0.009101** | 1.44 | OKAY |
| BAD | **0.45** | **0.034810** | **-2.21** | OKAY |
| BAG1 | **0.49** | **0.013568** | **-2.02** | OKAY |
| BAG3 | **6.79** | **0.000827** | **6.79** | OKAY |
| BAK1 | 1.60 | 0.095949 | 1.60 | OKAY |
| BAX | 0.55 | **0.000285** | -1.82 | OKAY |
| BCL10 | **4.30** | **0.000201** | **4.30** | OKAY |
| BCL2 | 0.78 | **0.015878** | -1.29 | OKAY |
| BCL2A1 | **63.84** | **0.001704** | **63.84** | A |
| BCL2L1 | **0.50** | **0.004967** | **-2.02** | OKAY |
| BCL2L10 | 1.34 | 0.543220 | 1.34 | B |
| BCL2L11 | **4.72** | **0.000331** | **4.72** | OKAY |
| BCL2L2 | **0.33** | **0.000040** | **-3.05** | OKAY |
| BFAR | 0.97 | 0.557900 | -1.03 | OKAY |
| BID | 0.97 | 0.590943 | -1.03 | OKAY |
| BIK | **12.81** | **0.000851** | **12.81** | OKAY |
| BIRC2 | 1.66 | **0.018825** | 1.66 | OKAY |
| BIRC3 | **0.20** | **0.000236** | **-5.12** | OKAY |
| BIRC5 | **0.38** | **0.002117** | **-2.64** | OKAY |
| BIRC6 | 0.59 | **0.003159** | -1.69 | OKAY |
| BNIP2 | 1.17 | 0.347136 | 1.17 | OKAY |
| BNIP3 | 0.78 | 0.050813 | -1.29 | OKAY |
| BNIP3L | 0.81 | **0.012460** | -1.23 | OKAY |
| BRAF | 0.95 | 0.716562 | -1.05 | OKAY |
| CASP1 | **0.43** | 0.205969 | **-2.35** | OKAY |
| CASP10 | 1.18 | 0.468126 | 1.18 | OKAY |
| CASP14 | **20.12** | **0.005966** | **20.12** | OKAY |
| CASP2 | **0.49** | **0.000242** | **-2.04** | OKAY |
| CASP3 | 1.99 | **0.005160** | 1.99 | OKAY |
| CASP4 | 0.93 | 0.553254 | -1.08 | OKAY |
| CASP5 | **9.01** | **0.003574** | **9.01** | OKAY |
| CASP6 | 0.58 | **0.000890** | -1.74 | OKAY |
| CASP7 | 0.78 | **0.046379** | -1.28 | OKAY |
| CASP8 | 0.70 | **0.014899** | -1.43 | OKAY |
| CASP9 | 1.27 | **0.013964** | 1.27 | OKAY |
| CD27 | **13.56** | **0.000473** | **13.56** | OKAY |
| CD40 | 1.71 | 0.054862 | 1.71 | OKAY |
| CD40LG | 1.38 | **0.034916** | 1.38 | OKAY |
| CD70 | **0.24** | **0.000356** | **-4.17** | OKAY |
| CFLAR | 0.67 | **0.042535** | -1.50 | OKAY |
| CIDEA | **13.37** | **0.006108** | **13.37** | A |
| CIDEB | 0.84 | 0.187223 | -1.19 | OKAY |
| CRADD | **0.22** | **0.047008** | **-4.64** | OKAY |
| CYCS | **4.92** | **0.000329** | **4.92** | OKAY |
| DAPK1 | **0.10** | **0.000014** | **-10.41** | OKAY |
| DFFA | 0.90 | **0.039248** | -1.11 | OKAY |
| DIABLO | 1.39 | 0.118760 | 1.39 | OKAY |
| FADD | 0.55 | **0.003518** | -1.82 | OKAY |
| FAS | 0.78 | 0.231071 | -1.28 | OKAY |
| FASLG | **5.25** | **0.001018** | **5.25** | OKAY |
| GADD45A | **22.28** | **0.028233** | **22.28** | OKAY |
| HRK | **24.39** | **0.016968** | **24.39** | OKAY |
| IGF1R | **0.13** | **0.000131** | **-7.78** | OKAY |
| IL10 | **12.48** | **0.001069** | **12.48** | A |
| LTA | **2.99** | **0.000716** | **2.99** | OKAY |
| LTBR | **0.36** | **0.000079** | **-2.79** | OKAY |
| MCL1 | **2.24** | **0.000014** | **2.24** | OKAY |
| NAIP | **0.38** | **0.002632** | **-2.60** | OKAY |
| NFKB1 | 0.63 | **0.013828** | -1.59 | OKAY |
| NOD1 | **0.41** | **0.000202** | **-2.43** | OKAY |
| NOL3 | 0.77 | 0.050434 | -1.29 | OKAY |
| PYCARD | **24.39** | **0.006158** | **24.39** | A |
| RIPK2 | 1.67 | **0.002664** | 1.67 | OKAY |
| TNF | **70.12** | **0.000179** | **70.12** | A |
| TNFRSF10A | 1.08 | 0.672878 | 1.08 | OKAY |
| TNFRSF10B | 1.38 | 0.103124 | 1.38 | OKAY |
| TNFRSF11B | 1.86 | 0.688535 | 1.86 | B |
| TNFRSF1A | 0.51 | **0.002077** | -1.96 | OKAY |
| TNFRSF1B | 1.86 | 0.275268 | 1.86 | B |
| TNFRSF21 | **2.16** | **0.009567** | **2.16** | OKAY |
| TNFRSF25 | **5.18** | 0.086918 | **5.18** | B |
| TNFRSF9 | **33.25** | **0.000554** | **33.25** | A |
| TNFSF10 | **0.34** | **0.001349** | **-2.92** | OKAY |
| TNFSF8 | **6.63** | 0.093110 | **6.63** | A |
| TP53 | 0.58 | **0.000961** | -1.72 | OKAY |
| TP53BP2 | 1.60 | **0.013676** | 1.60 | OKAY |
| TP73 | 1.34 | 0.088079 | 1.34 | OKAY |
| TRADD | 1.15 | 0.204464 | 1.15 | OKAY |
| TRAF2 | **0.36** | **0.001225** | **-2.81** | OKAY |
| TRAF3 | 0.66 | **0.002630** | -1.51 | OKAY |
| XIAP | 0.90 | 0.072969 | -1.11 | OKAY |
| ACTB | **0.48** | **0.000897** | **-2.07** | OKAY |
| B2M | 0.68 | 0.051404 | -1.48 | OKAY |
| GAPDH | 1.02 | 0.221209 | 1.02 | OKAY |
| HPRT1 | **0.49** | **0.000214** | **-2.03** | OKAY |
| RPLP0 | 0.98 | 0.218795 | -1.02 | OKAY |

**Supplementary Table 9: Lists of changes in relative expression for apoptosis genes in the Hep-G2 cells after treated with 3 (14 μM) for 24 h. The table lists genes that exhibit a difference in expression in the Hep-G2 cells sample when compared to control.**

| **Symbol** | **FoldChange** | **p value** | **Fold up/down-regulation** | **Comments** |
| --- | --- | --- | --- | --- |
| ABL1 | 1.14 | 0.100535 | 1.14 | OKAY |
| AIFM1 | 1.39 | **0.000130** | 1.39 | OKAY |
| AKT1 | 0.67 | **0.022126** | -1.50 | OKAY |
| APAF1 | 0.92 | 0.574755 | -1.08 | OKAY |
| BAD | 0.81 | 0.320187 | -1.23 | OKAY |
| BAG1 | **0.27** | **0.003869** | **-3.76** | OKAY |
| BAG3 | 1.12 | 0.343228 | 1.12 | OKAY |
| BAK1 | **2.10** | **0.012918** | **2.10** | OKAY |
| BAX | 1.03 | 0.710213 | 1.03 | OKAY |
| BCL10 | 1.24 | 0.163238 | 1.24 | OKAY |
| BCL2 | 1.08 | 0.504907 | 1.08 | OKAY |
| BCL2A1 | **15.30** | **0.000139** | **15.30** | A |
| BCL2L1 | 0.51 | **0.002463** | -1.97 | OKAY |
| BCL2L10 | 1.21 | 0.719208 | 1.21 | B |
| BCL2L11 | **2.71** | **0.001241** | **2.71** | OKAY |
| BCL2L2 | 1.01 | 0.769456 | 1.01 | OKAY |
| BFAR | 1.36 | **0.002483** | 1.36 | OKAY |
| BID | 0.80 | 0.263682 | -1.24 | OKAY |
| BIK | **3.02** | 0.083191 | **3.02** | OKAY |
| BIRC2 | 1.65 | **0.006781** | 1.65 | OKAY |
| BIRC3 | 0.56 | **0.002221** | -1.79 | OKAY |
| BIRC5 | 0.77 | 0.063105 | -1.29 | OKAY |
| BIRC6 | 1.22 | 0.062768 | 1.22 | OKAY |
| BNIP2 | 1.48 | **0.040196** | 1.48 | OKAY |
| BNIP3 | 1.82 | 0.084516 | 1.82 | OKAY |
| BNIP3L | **2.06** | **0.011494** | **2.06** | OKAY |
| BRAF | 1.10 | 0.244362 | 1.10 | OKAY |
| CASP1 | 1.61 | 0.346283 | 1.61 | OKAY |
| CASP10 | 0.69 | **0.042010** | -1.45 | OKAY |
| CASP14 | **8.51** | **0.049187** | **8.51** | OKAY |
| CASP2 | 0.83 | 0.169179 | -1.21 | OKAY |
| CASP3 | 1.46 | **0.003465** | 1.46 | OKAY |
| CASP4 | 1.39 | **0.019791** | 1.39 | OKAY |
| CASP5 | 1.47 | **0.028310** | 1.47 | C |
| CASP6 | 1.46 | **0.019568** | 1.46 | OKAY |
| CASP7 | 1.23 | 0.099618 | 1.23 | OKAY |
| CASP8 | 1.13 | 0.363521 | 1.13 | OKAY |
| CASP9 | **3.47** | **0.000267** | **3.47** | OKAY |
| CD27 | **4.42** | **0.000453** | **4.42** | OKAY |
| CD40 | 0.92 | 0.890806 | -1.09 | OKAY |
| CD40LG | 1.16 | 0.405531 | 1.16 | OKAY |
| CD70 | 0.62 | **0.011373** | -1.62 | OKAY |
| CFLAR | 1.19 | **0.041039** | 1.19 | OKAY |
| CIDEA | **2.51** | 0.084741 | **2.51** | B |
| CIDEB | 0.75 | 0.137486 | -1.33 | OKAY |
| CRADD | 1.01 | 0.827678 | 1.01 | OKAY |
| CYCS | **2.27** | **0.023384** | **2.27** | OKAY |
| DAPK1 | 0.82 | 0.388965 | -1.22 | OKAY |
| DFFA | 1.10 | 0.190875 | 1.10 | OKAY |
| DIABLO | 1.68 | **0.017469** | 1.68 | OKAY |
| FADD | 1.24 | 0.233431 | 1.24 | OKAY |
| FAS | 0.69 | 0.103018 | -1.45 | OKAY |
| FASLG | 1.37 | 0.256212 | 1.37 | OKAY |
| GADD45A | **3.98** | **0.002504** | **3.98** | OKAY |
| HRK | **49.72** | **0.000620** | **49.72** | OKAY |
| IGF1R | **0.40** | **0.000683** | **-2.51** | OKAY |
| IL10 | 1.11 | 0.619642 | 1.11 | B |
| LTA | 0.71 | 0.303102 | -1.40 | OKAY |
| LTBR | 0.81 | **0.019868** | -1.24 | OKAY |
| MCL1 | **2.15** | **0.003151** | **2.15** | OKAY |
| NAIP | 0.95 | 0.657086 | -1.05 | OKAY |
| NFKB1 | 0.54 | **0.001678** | -1.85 | OKAY |
| NOD1 | 0.77 | 0.064865 | -1.30 | OKAY |
| NOL3 | 0.93 | 0.387902 | -1.07 | OKAY |
| PYCARD | **2.80** | **0.011438** | **2.80** | A |
| RIPK2 | 1.45 | **0.000054** | 1.45 | OKAY |
| TNF | **16.66** | **0.027907** | **16.66** | A |
| TNFRSF10A | 0.98 | 0.993929 | -1.02 | OKAY |
| TNFRSF10B | 1.11 | 0.305094 | 1.11 | OKAY |
| TNFRSF11B | **3.22** | 0.204192 | **3.22** | A |
| TNFRSF1A | 0.94 | 0.483282 | -1.06 | OKAY |
| TNFRSF1B | 0.96 | 0.717467 | -1.05 | B |
| TNFRSF21 | **3.02** | **0.000025** | **3.02** | OKAY |
| TNFRSF25 | **2.68** | 0.068413 | **2.68** | B |
| TNFRSF9 | **110.29** | 0.062216 | **110.29** | A |
| TNFSF10 | 1.70 | 0.333877 | 1.70 | OKAY |
| TNFSF8 | **3.25** | **0.017895** | **3.25** | OKAY |
| TP53 | 0.95 | 0.642674 | -1.05 | OKAY |
| TP53BP2 | 1.53 | **0.004923** | 1.53 | OKAY |
| TP73 | 1.38 | 0.254696 | 1.38 | OKAY |
| TRADD | 1.11 | 0.531569 | 1.11 | OKAY |
| TRAF2 | 0.82 | 0.100000 | -1.21 | OKAY |
| TRAF3 | 1.01 | 0.910578 | 1.01 | OKAY |
| XIAP | 1.26 | 0.067174 | 1.26 | OKAY |
| ACTB | **0.31** | **0.000216** | **-3.23** | OKAY |
| B2M | 1.32 | 0.217504 | 1.32 | OKAY |
| GAPDH | 0.86 | 0.171918 | -1.17 | OKAY |
| HPRT1 | 0.54 | **0.000448** | -1.87 | OKAY |
| RPLP0 | 1.17 | 0.210834 | 1.17 | OKAY |
